# LRP10 interacts with SORL1 in the intracellular vesicle trafficking pathway in non-neuronal brain cells and localises to Lewy bodies in Parkinson’s disease and dementia with Lewy bodies

**DOI:** 10.1007/s00401-021-02313-3

**Published:** 2021-04-28

**Authors:** Martyna M. Grochowska, Ana Carreras Mascaro, Valerie Boumeester, Domenico Natale, Guido J. Breedveld, Hanneke Geut, Wiggert A. van Cappellen, Agnita J. W. Boon, Anneke J. A. Kievit, Esther Sammler, Netherlands Brain Bank, Piero Parchi, Pietro Cortelli, Dario R. Alessi, Wilma D. J. van de Berg, Vincenzo Bonifati, Wim Mandemakers

**Affiliations:** 1 Department of Clinical Genetics, Erasmus MC, University Medical Center Rotterdam, P.O. Box 2040, 3000 CA Rotterdam, The Netherlands; 2 Department of Anatomy and Neurosciences, Amsterdam Neuroscience, Amsterdam UMC, Vrije Universiteit Amsterdam, P.O. Box 7057, 1007 MB Amsterdam, The Netherlands; 3 Netherlands Institute for Neuroscience, Meibergdreef 47, 1105 BA Amsterdam, The Netherlands; 4 Erasmus Optical Imaging Centre (OIC), Erasmus MC, University Medical Center Rotterdam, P.O. Box 2040, 3000 CA Rotterdam, The Netherlands; 5 Department of Neurology, Erasmus MC, University Medical Center Rotterdam, P.O. Box 2040, 3000 CA Rotterdam, The Netherlands; 6 Medical Research Council (MRC) Protein Phosphorylation and Ubiquitylation Unit, School of Life Sciences, University of Dundee, Dundee, DD1 5EH UK; 7 Department of Neurology, School of Medicine, Ninewells Hospital, University of Dundee, Dundee, DD1 9SY UK; 8 Istituto di Ricovero e Cura a Carattere Scientifico (IRCCS), Istituto di Scienze Neurologiche di Bologna, Via Altura 3, 40139 Bologna, Italy; 9 Department of Experimental, Diagnostic and Specialty Medicine (DIMES), University of Bologna, Via Massarenti 9, 40138 Bologna, Italy; 10 Dipartimento di Scienze Biomediche e NeuroMotorie (DIBINEM), Alma Mater Studiorum-University of Bologna, Via Altura 3, 40139 Bologna, Italy

**Keywords:** LRP10, Parkinson’s disease (PD), Dementia with Lewy bodies (DLB), Vesicle trafficking, Astrocytes, Lewy bodies

## Abstract

Loss-of-function variants in the low-density lipoprotein receptor-related protein 10 (*LRP10*) gene have been associated with autosomal-dominant Parkinson’s disease (PD), PD dementia, and dementia with Lewy bodies (DLB). Moreover, *LRP10* variants have been found in individuals diagnosed with progressive supranuclear palsy and amyotrophic lateral sclerosis. Despite this genetic evidence, little is known about the expression and function of LRP10 protein in the human brain under physiological or pathological conditions. To better understand how *LRP10* variants lead to neurodegeneration, we first performed an in-depth characterisation of LRP10 expression in post-mortem brains and human-induced pluripotent stem cell (iPSC)-derived astrocytes and neurons from control subjects. In adult human brain, LRP10 is mainly expressed in astrocytes and neurovasculature but undetectable in neurons. Similarly, LRP10 is highly expressed in iPSC-derived astrocytes but cannot be observed in iPSC-derived neurons. In astrocytes, LRP10 is present at trans-Golgi network, plasma membrane, retromer, and early endosomes. Interestingly, LRP10 also partially co-localises and interacts with sortilin-related receptor 1 (SORL1). Furthermore, although LRP10 expression and localisation in the substantia nigra of most idiopathic PD and DLB patients and *LRP10* variant carriers diagnosed with PD or DLB appeared unchanged compared to control subjects, significantly enlarged LRP10-positive vesicles were detected in a patient carrying the LRP10 p.Arg235Cys variant. Last, LRP10 was detected in Lewy bodies (LB) at late maturation stages in brains from idiopathic PD and DLB patients and in *LRP10* variant carriers. In conclusion, high LRP10 expression in non-neuronal cells and undetectable levels in neurons of control subjects indicate that LRP10-mediated pathogenicity is initiated via cell non-autonomous mechanisms, potentially involving the interaction of LRP10 with SORL1 in vesicle trafficking pathways. Together with the specific pattern of LRP10 incorporation into mature LBs, these data support an important mechanistic role for disturbed vesicle trafficking and loss of LRP10 function in neurodegenerative diseases.

**Supplementary Information:**

The online version contains supplementary material available at 10.1007/s00401-021-02313-3.

## Introduction

Parkinson’s disease (PD) and dementia with Lewy bodies (DLB) are common neurodegenerative disorders strongly affecting the ageing population worldwide [27, 85]. Cardinal motor features of PD include bradykinesia, rigidity and rest tremor [12, 69]. These motor signs are usually preceded by non-motor manifestations such as olfactory dysfunction, rapid eye movement (REM) sleep behaviour disorder, depression, and constipation [12, 69]. Additionally, over the course of the disease, cognitive impairment also develops with progression to dementia in up to 80% of the patients [1, 36, 52]. DLB is clinically defined by a gradual cognitive decline as the initial manifestation, followed by parkinsonism in over 85% of cases [59]. Although PD and DLB differ in the temporal sequence of prominent clinical symptoms, both are neuropathologically characterised by neuronal loss and the formation of intracellular α-synuclein-containing inclusions in surviving neurons, termed Lewy bodies (LB) and Lewy neurites (LN) [31].

In addition to overlapping clinical and neuropathological features, rare, highly penetrant pathogenic variants in *SNCA* (α-synuclein) [43, 68, 92] and *LRRK2* (leucine-rich repeat kinase 2) [65, 94] have been identified in patients with phenotypes ranging from familial PD to DLB. Furthermore, heterozygous variants in *GBA* (glucocerebrosidase) are a strong risk factor for the development of both PD and DLB [61, 77]. The recently discovered pathogenic variants in *LRP10* (low-density lipoprotein receptor-related protein 10) associated with autosomal-dominant, inherited forms of PD, PD dementia (PDD) and DLB, further strengthen the evidence of the overlapping genetic bases in the Lewy body disorders (LBD) [70, 86]. Moreover, the association of LRP10 variants with progressive supranuclear palsy (PSP) [87] and amyotrophic lateral sclerosis (ALS) [63], together with the data showing that LRP10 is a driver of a specific molecular subtype of Alzheimer’s disease (AD) [62], provide evidence of potential mechanistic roles for LRP10 across a broader spectrum of neurodegenerative diseases.

The physiological roles of LRP10 and how its defects contribute to the pathogenesis of these major neurodegenerative disorders associated with parkinsonism and dementia remain mostly unknown. Previous studies using overexpression models linked LRP10 to vesicle trafficking, showing its localisation at the plasma membrane, endosomes, trans-Golgi network, and a partial overlap with the retromer complex [15, 17, 18, 26, 70]. However, the cellular and subcellular localisation of endogenous LRP10 protein in the central nervous system (CNS) during normal ageing or disease has not yet been studied.

To gain more insight into the physiological and pathological role of LRP10 and how its loss-of-function is mechanistically involved in the pathogenesis of PD, PDD, and DLB, we performed an in-depth characterisation of endogenous LRP10 expression in human-induced pluripotent stem cell (iPSC)-derived astrocytes and neurons, and human post-mortem brains from control subjects, idiopathic PD and DLB cases, and *LRP10* variant carriers diagnosed with PD or DLB, by biochemical analyses and detailed multi-label laser scanning confocal microscopy using knockout (KO) validated LRP10 antibodies targeting different LRP10 protein domains [70, 86]. In addition to a detailed description of LRP10 localisation in the ageing and diseased brain, our study also provides evidence for a cell non-autonomous role for LRP10 in disease pathogenesis.

## Materials and methods

### Study cases

We included post-mortem brain tissue specimens from 28 donors. The demographics, clinical, and neuropathological characteristics of all donors are listed in the Online Resource Table 1. Brain tissue specimens from 27 donors were provided by the Netherlands Brain Bank (NBB), Netherlands Institute for Neuroscience, Amsterdam. Brain specimens from Patient-III were obtained from the Laboratory of Neuropathology—the Institute of Neurological Sciences of Bologna (ISNB), Italy. Ethical approval for the NBB procedures and forms was given by the Medical Ethics Committee of the VU University Medical Center (Amsterdam, the Netherlands). Autopsies are performed by the NBB at the designated premises of the VU Medical Center in Amsterdam, the Netherlands. Autopsy procedures and tissue collection at both centres were performed in accordance with the Code of Conduct for Brain Banking [41] and Declaration of Helsinki (1964 and its later amendments). For both centres, informed consent for brain autopsy, and the use of the tissue and clinical information for research purposes was obtained from all individual participants included in this study or from the next of kin. Clinical and pathological features of a total of five donors carrying rare, pathogenic variants in *LRP10* included in this study are summarized in the Online Resource Table 1. Briefly, patient I carrying a heterozygous *LRP10* c.451C>T (p.Arg151Cys) variant was clinically diagnosed with AD and showed intermediate AD-type pathology and cortical Lewy pathology fitting with DLB upon neuropathological examination [86]. A heterozygous *LRP10* variant c.632dupT (p.Ala212Ser fs*17) was identified in patient II who was clinically diagnosed with PD and mild cognitive impairment, and on neuropathological examination showed Lewy pathology and mild AD-type pathology [70]. A heterozygous *LRP10* variant c.703C>T (p.Arg235Cys) was identified in patient III who was clinically diagnosed with PDD and on neuropathological examination showed Lewy pathology and mild AD-type pathology [70]. Patient IV is a carrier of a *LRP10* c.1357G>A (p.Gly453Ser) and was clinically diagnosed with DLB and designated as mixed AD/LBD upon neuropathological diagnosis [86]. Finally, an in-frame deletion in LRP10, c.1549_1551delAAT (p.Asn517del) was identified in patient V who was clinically and neuropathologically diagnosed with DLB and showed intermediate AD-pathology [70].

### Primary and secondary antibodies

Details of all commercial primary antibodies used in this study are listed in the Online Resource Table 2. Polyclonal sheep anti-LRP10 (LRP10-CT) was generated in collaboration with Medical Research Council (MRC) Protein Phosphorylation and Ubiquitylation Unit Reagents, University of Dundee (MRC PPU Reagents, DA058). Secondary antibodies used for immunocytochemistry and immunohistochemistry include: Alexa Fluor^®^ 488 donkey anti-rabbit/mouse/sheep/goat; Alexa Fluor^®^ 594 donkey anti-rabbit/mouse, Alexa Fluor^®^ 647 goat anti-guinea pig (all from Jackson ImmunoResearch Laboratories). Secondary antibodies used for Western blotting include: IRDye^®^ 680 donkey anti-mouse/rabbit (LI-COR Biosciences) and Alexa Fluor^®^ donkey anti-sheep (Jackson ImmuneResearch Laboratories).

### Cloning

To clone LRP10 expression construct, the MGC human LRP10 sequence-verified cDNA was subcloned from the human LRP10-pCR4-TOPO plasmid (Dharmacon, 8322768) into the pcDNA™3·1/V5-His-TOPO^®^ with a stop codon interrupting the expression of the existing V5 tag. Another V5 tag was introduced into the N-terminal position relative to the LRP10 insert.

For SORL1 cloning, the human SORL1 sequence-verified cDNA was subcloned from the SORL1-pCR-XL-TOPO plasmid (Source bioscience, 9021168) into the pcDNA™3·1/V5-His-TOPO^®^ replacing the existing V5 tag. All expression constructs were verified by Sanger sequencing.

### Cell culture

HEK293T cells (ATCC^®^ CRL-3216™) were maintained in growth medium [DMEM (Lonza), 10% (v/v) FBS (Sigma-Aldrich)] at 37 °C/5%CO_2_. HuTu 80 cells (ATCC^®^ HTB-40™) were maintained in growth medium [DMEM/F-12 (Gibco), 10% (v/v) FBS] at 37 °C/5% CO_2_.

### CRISPR/Cas9 LRP10 knockout in HEK293T and HuTu 80 cells

For CRISPR/Cas9-mediated KO, 20 bp single guide RNA (sgRNA) targeting *LRP10* exon 1 was selected using CHOPCHOP web tool (http://chopchop.cbu.uib.no) [46]. sgRNA with the highest score was cloned into the pSpCas9-(BB)-2A-GFP (Addgene, 48138) plasmid. Briefly, sense and antisense oligonucleotides (5′-P-CACCGCGTTTCGGTTCTTACCAAGG & 5′-P-AAACCCTTGGTAAGAACCGAAACGG, Integrated DNA Technologies) with BbsI overhangs were synthesized and annealed. Annealed oligonucleotides were cloned into the BbsI (NEB) digested pSpCas9-(BB)-2A-GFP plasmid. All constructs were verified by Sanger sequencing. Cells were transfected with the pSpCas9-(BB)-2A-GFP plasmid containing sgRNA using GeneJuice transfection reagent (Merck) according to the manufacturer’s specifications. After 48 h, cells were dissociated with Trypsin–EDTA (Gibco). GFP-positive cells were sorted as single cells into 96-well plates with the BD FACSAria™ III cell sorter. Recovered clones were expanded as independent clones and genotyped. Primers flanking *LRP10* exon 1 (FW: CAAAGTTTGGCCCGAAGAGG, RV: GGGCAGGCAGGATAGAGTGC) were used for PCR amplification. PCR products were Sanger sequenced and analysed for the presence of INDELs.

### Generation and characterisation of human iPSC lines

The control line was derived from dermal fibroblasts of a healthy male donor at the age of 57. These cells were reprogrammed and characterised as described earlier [70, 83]. All study procedures were approved by the medical ethical committee of Erasmus MC, conformed to the principles of the WMA Declaration of Helsinki and the Department of Health and Human Services Belmont report. Participating subjects provided written informed consent for the use of the material for research purposes.

### Generation of neural progenitor cells and differentiation into ventral midbrain dopaminergic neurons and astrocytes

Neural progenitor cells were created via inhibition of both BMP and TGFβ signalling (SMAD) and stimulation of canonical WNT and SHH signalling pathways in free floating embryoid bodies according to published protocols [71] with minor modifications as described previously [70]. Briefly, iPSC were detached from mouse embryonic fibroblasts (MEF) using 2 mg/mL collagenase IV (Gibco). Pieces of colonies were resuspended in human Embryonic Stem Cell medium [hESC, 80% DMEM/F-12, 20% KnockOut Serum Replacement, 1% l-glutamine, 1% Penicilin-Streptomycin, 1% MEM-NEAA (all from Thermo Fisher Scientific), and 0.0007% 2-β-Mercaptoethanol (Sigma-Aldrich)] supplemented with 10 µM SB-431542 (Tocris), 1 µM dorsomorphin (Abcam), 3 µM CHIR99021 (CHIR, Sigma), and 0.5 µM Purmorphamine (PMA, Stem Cell Technologies). Clumps of cells were transferred to 10 cm Petri dishes and were cultured in suspension for a total of 6 days on a shaker at 80 RPM at 37 °C/5% CO_2_. On day 2, medium was replaced by N2B27 medium [DMEM/F-12 – Neurobasal in 1:1 ratio, 1:100 B27 w/o Vitamin A, 1:200 N2, 1% Penicilin-Streptomycin (all from Thermo Fisher Scientific)] supplemented with 10 µM SB-431542, 1 µM dorsomorphin, 3 µM CHIR, and 0.5 µM PMA. On day 4, medium was changed to N2B27 supplemented with 3 µM CHIR, 0.5 µM PMA, and 150 µM Ascorbic Acid (AA, Sigma). On day 6, embryoid bodies showing neuroepithelial development were selectively taken up, triturated into smaller pieces, and plated on Corning^®^ Matrigel^®^ coated 12-well plates in N2B27 medium supplemented with 3 µM CHIR, 200 µM AA, and 0.5 µM PMA. Cell splits were performed at 1:10 ratio. From the sixth passage, 0.5 µM PMA was replaced by 0.5 µM Smoothened Agonist (SAG, Abcam). Cells were passaged at least five times before final differentiations. For ventral midbrain neuronal differentiation, neural progenitors were dissociated with accutase (Sigma) at room temperature (RT), resuspended, and plated (50.000 cells/well) on Corning^®^ Matrigel^®^ and 0.1 mg/mL poly-d-lysine (Sigma) coated 18 mm glass coverslips in 12-well plates in N2B27 medium supplemented with 1 ng/mL GDNF (PeproTech), 2 ng/mL BDNF (PeproTech), 200 µM AA, and 0.5 µM SAG for a duration of 6 days. Medium was changed every other day. After 6 days, cells were refreshed with N2B27 medium containing 1 ng/mL GDNF, 2 ng/mL BDNF, 200 µM AA, 1 ng/mL TGF-β3, 5 ng/mL ActivinA (Stem Cell Technologies). ActivinA concentration was lowered to 2 ng/mL for the subsequent feedings. Medium was changed every other day. Neuronal cultures reached mature stage after 3 weeks of differentiation. For directed astrocyte differentiation, cells were cultured for at least 8 weeks. Briefly, neural progenitors were cultured on Corning^®^ Matrigel^®^ coated 12-well plates for 2 days in N2B27 medium supplemented with 10 ng/mL FGF-basic (PeproTech) and 10 ng/mL EGF (PeproTech). After 2 days, this medium was switched to N2 medium (Advanced DMEM/F-12, 1% N2, 1% Penicilin-Streptomycin, 4% FBS) supplemented with 10 ng/mL CNTF (PeproTech) for 2 weeks. Throughout the whole differentiation procedure, astrocyte cultures were split using accutase when they reached 80–90% confluence. After CNTF withdrawal, cells were expanded for several weeks in N2 medium with 10 ng/mL EGF. One week before terminating cultures, cells were treated with 500 µM dbcAMP in N2 medium.

### RNA extraction from cultured cells and brain tissue

Total RNA from cultured cells was isolated using the RNeasy Mini Kit (Qiagen) as recommended by the manufacturer. Total RNA from substantia nigra of non-demented controls (*N* = 6, Online Resource Table 1, NDC VI-XI) and PD patients (*N* = 6, Online Resource Table 1, PD IV-IX) was isolated using a combination of TRIzol™ (Sigma-Aldrich) and column isolation protocols. Briefly, TRIzol™ was added to substantia nigra pieces that were cut using cryostat from frozen at autopsy brain tissues. Tissue was disrupted using rotor–stator homogenizer and then incubated 5 min at RT. After incubation, chloroform (Sigma) was added, and the tube was shaken vigorously by hand for 15 s. The mix was incubated for 15 min at RT and subsequently centrifuged at 13,000 RPM at 4 °C. Clear, upper aqueous phase was collected and mixed in 0.53 volume of RNAse-free 70% ethanol. The mix was incubated for 5 min at RT. The samples were then transferred to the HighBind™ RNA Mini Column (Omega BIO-TEK). The isolation was proceeded according to the E.Z.N.A. Total RNA kit I—Animal Tissue Protocol (Omega BIO-TEK). For each sample, on-membrane DNase I (RNase-Free DNase Set, Qiagen) digestion was performed according to the manufacturer’s protocol. The integrity of the total RNA was assessed using agarose gel stained with GelRed™ (Biotium). Sharp, clear 28S and 18S rRNA bands without smearing indicated intact RNA. Spectrophotometric analysis of extracted RNA using NanoDrop 2000/2000c measured 260/280 values of ~ 2.0.

### Reverse transcription and quantitative PCR

For the cDNA synthesis, 0.5 µg of the total RNA was used with random hexamers using SuperScript^®^ III First-Strand Synthesis System (Thermo Fisher Scientific) followed by RNase H digestion. qPCR using iTaq Universal SYBR Green Supermix (Bio-Rad) was performed using ~ 100 ng cDNA with the following cycling conditions: 3 min at 95 °C (initial denaturation), 40 cycles of 5 s at 95 °C, and 30 s at 60 °C. Data analysis was performed using CFX Manager™ software 3.0 (Bio-Rad). Briefly, the normalized expression of each target gene was calculated using the delta–delta *C*_q_ method [54]. Relative *LRP10* mRNA levels were determined after normalisation to the geometric mean of the following housekeeping genes: *COPS5*, *CLK2*, and *RNF10*. Primers were designed using Primer3 (v. 0.4.0) online tool (https://bioinfo.ut.ee/primer3-0.4.0/) and are listed in the Online Resource Table 3.

### Western blotting

Protein lysates were obtained after washing the cells with PBS and subsequently adding protein lysis buffer [50 mM Tris–Cl (pH 7.4), 100 mM NaCl, 1.0% IGEPAL^®^ CA-630 (all from Sigma-Aldrich)] containing protease inhibitors Complete^®^ and Pefabloc^®^ SC (both from Merck). Lysates were snap frozen, thawed on ice and cleared by centrifugation at 13,000 RPM for 10 min at 4 °C. Lysates were mixed with 4 × sample buffer [8% SDS, 20% v/v glycerol, 0.002% bromophenolblue, 62.5 mM Tris–Cl (pH 6.8)] supplemented with 100 mM dithiothreitol (DTT) and incubated for 10 min at 95 °C. Proteins were separated on 4–15% Criterion TGX precast gels (Bio-Rad), and transferred to nitrocellulose membranes using the Trans-Blot^®^ Turbo™ Transfer System (Bio-Rad). Blots were blocked using 5% non-fat dry milk (Blotto, Santa Cruz Biotechnologies) in PBS for 1 h at RT. Primary antibody incubations were performed overnight at 4 °C in blocking buffer. After washing in PBS, 0.1% v/v TWEEN^®^ 20, blots were incubated for 1 h at RT with fluorescently conjugated secondary antibodies (LI-COR Biosciences or Jackson ImmuneResearch). After washing in PBS, 0.1% v/v TWEEN^®^ 20, blots were imaged using the Odyssey CLx Imaging system and analysed with Image Studio™ Lite Ver 5.2 (both from LI-COR Biosciences).

### Co-immunoprecipitation

HEK293T cell lines were plated on 10 cm dishes and transfected with V5-tagged LRP10 and SORL1 expression constructs using GeneJuice^®^ transfection reagent according to manufacturer’s specifications. After 48 h, protein lysates were collected, and protein concentrations were determined via Pierce™ BCA Protein Assay Kit (Thermo Fisher Scientific). For co-immunoprecipitation, 20 μL of Pierce™ Protein G Magnetic Beads (Thermo Fisher Scientific) were washed three times with washing buffer [50 mM Tris–Cl (pH 7.4), 0.5 M NaCl, 0.05% v/v TWEEN^®^ 20] and incubated with 400 μg of protein lysate in a final volume of 600 μL for 1 h at 4 °C. Beads were magnetized and discarded. 20 μL of fresh beads were washed and incubated with 2 μg of antibody for 10 min at RT. Beads were washed three times with washing buffer and incubated with precleared protein lysates overnight at 4 °C. Beads were washed three times with washing buffer supplemented with 0.05% v/v IGEPAL^®^ CA-630 and eluted with 10 μL of 2 × sample buffer [4% SDS, 10% v/v glycerol, 0.001% bromophenolblue, 62.5 mM Tris–Cl (pH 6.8)] supplemented with 100 mM dithiothreitol (DTT) for 10 min at 95 °C. Samples were analysed by Western blotting. Antibodies used for pulldown: mouse anti-SORL1 (BD Biosciences, 612633), sheep anti-LRP10 (MRC PPU Reagents, DA058), and Mouse Gamma Globulin (Jackson ImmunoResearch Laboratories, 015-000-002) used as a negative control in place of a primary antibody to evaluate any non-specific binding.

### Immunocytochemistry on iPSCs

Cells were fixed with 4% paraformaldehyde (Sigma-Aldrich) for 10 min, and then washed with PBS. After washing, cells were incubated in a blocking buffer [50 mM Tris.HCl (pH 7.4), 0.9% NaCl, 0.25% gelatine, 0.2% Triton™ X-100] containing primary antibody overnight at 4 °C. Next day, cells were washed with PBS buffer containing 0.05% TWEEN^®^ 20. Cells were then incubated for 1 h with appropriate Alexa Fluor^®^ antibodies (all Jackson ImmunoResearch Laboratories) at RT. After washing off secondary antibodies with PBS-TWEEN^®^ 20 buffer, cells were mounted with ProLong Gold with DAPI (Invitrogen). To fluorescently label endogenous LRP10 at the plasma membrane, 10-week-old iPSC-derived astrocytes were washed twice with ice-cold DMEM and subsequently incubated for 2 h on ice with ice-cold DMEM (Gibco) containing rabbit anti-LRP10 antibody (Sino Biological, 13228-T16). Next, cells were washed twice with ice-cold DMEM, fixed with 4% paraformaldehyde, and processed for staining with Alexa Fluor^®^ 488 antibody. Secondary only was used as a negative control. All stainings were imaged with Leica SP5 AOBS confocal microscope. Scanning was done with a pixel size of 0.12 µm and with a scan size of 2048 × 2048 pixels at 400 Hz. For z-stack images, 0.35 µm steps in the z-direction were taken. The pinhole size was set to 1 airy unit (AU). Sections were irradiated with the following lasers: 405 diode UV, argon laser, DPSS 561, HeNe 633, depending on fluorophore combination. Each image was detected on the spectral PMT detector with an HCX PL APO CS 40 ×/1.25 or HCX PL APO CS 63 ×/1.4 lens.

### Immunohistochemistry on brain specimens

Immunohistochemistry on formalin-fixed, paraffin-embedded (FFPE) human brain samples was carried on 6–8 μm paraffin-embedded tissue sections from different brain regions (substantia nigra, medial frontal gyrus (F2), temporal pole, hippocampus). Sections were deparaffinized in xylene (Merck) for a total of 10 min and rehydrated in graded series of ethanol. Heat-induced antigen retrieval in a pressure cooker was applied to slides destined for LRP10 (Sino Biological, 13228-T16) staining. Slides were boiled in 10 mM sodium citrate buffer (pH 6.0) in autoclave at 121 °C for 20 min. To block endogenous peroxidase activity, tissue sections were quenched with PBS with 0.6% H_2_O_2_ for 30 min. Subsequently, sections were incubated with a blocking buffer [PBS, 5% milk (w/v, Nutricia)] for 30 min. Tissue sections were stained overnight at 4 °C with rabbit anti-LRP10 (SinoBiological, 13228-T16). Next day, sections were washed three times with PBS-0.1% TWEEN^®^ 20. Sections were then incubated with polyHRP secondary antibody (polyHRP anti Ms/Rb IgG, BrightVision) at RT for 1 h. To fluorescently label LRP10, sections were incubated for 4 min at RT with 1:50 dilution of tetramethylrhodamine–tyramide in TSA PLUS amplification buffer (all Perkin-Elmer). Sections were immediately washed with PBS-0.1% TWEEN^®^ 20. Endogenous fluorescence was quenched with 0.1% (w/v) Sudan Black B (Sigma) in 70% ethanol for 5 min. For double labelling with various cellular markers, sections were exposed again to an antigen retrieval method as recommended by antibody manufactures and stained with a primary antibody overnight 4 °C. Sections were washed with PBS-0.1%TWEEN^®^ 20 buffer and incubated with an appropriate secondary Alexa Fluor^®^ antibodies (all from Jackson ImmunoResearch Laboratories) for 1 h at RT. After washing off secondary antibodies with PBS-0.1% TWEEN^®^ 20 buffer, sections were mounted with ProLong Gold with DAPI. Scanning was done with a pixel size of 0.12 µm and with a scan size of 2048 × 2048 pixels at 400 Hz. For z-stack images, 0.35 µm steps in the z-direction were taken. The pinhole size was set to 1 airy unit (AU). Sections were irradiated with the following lasers: 405 diode UV, argon laser, DPSS 561, HeNe 633, depending on fluorophore combination. Each image was detected on the spectral PMT detector with an HCX PL APO CS 40 ×/1.25 or HCX PL APO CS 63 ×/1.4 lens.

### LRP10 and α-synuclein double labelling using immunohistochemistry on FFPE brain specimens

After deparaffinization and rehydration, tissue sections were first exposed to 80% formic acid (Sigma) for 5 min, permeabilized, blocked as mentioned above, and incubated with 1:100 mouse monoclonal anti-α-synuclein (clone 42/α-Synuclein, BD Bioscience, 610786) overnight at 4 °C. Sections were then incubated with polyHRP secondary antibody (polyHRP anti Ms/Rb IgG, BrightVision) at RT for 1 h. To fluorescently label α-synuclein, sections were incubated for 4 min at RT with 1 in 50 fluorescein-tyramide in TSA PLUS amplification buffer (all Perkin-Elmer). To label LRP10 and to strip α-synuclein primary antibody, sections were then exposed again to heat-induced antigen retrieval in Tris–EDTA buffer (10 mM Tris Base, 1 mM EDTA, 0.05% TWEEN^®^ 20, pH 9.0) and incubated with 1:100 sheep anti-LRP10 (MRC PPU Reagents, DA058) overnight at 4 °C. Sections were washed with PBS-TWEEN^®^ 20 buffer and incubated with donkey anti-sheep Alexa Fluor^®^ 647 (Jackson ImmunoResearch Laboratories) for 2 h at RT. Stainings were imaged with Leica SP5 AOBS confocal microscope (Leica Microsystems). Each image was detected on the spectral PMT detector with an HCX PL APO CS 40x/1.25 or HCX PL APO CS 63x/1.4 lens. For 3D volume renderings, stainings were imaged with STELLARIS 5 confocal microscope (Leica Microsystems) using a built-in LIGHTNING detection technology for super-resolution confocal imaging. Scanning was done with a pixel size of 0.029 µm with a scan size of 1080 × 1080 pixels at 400 Hz. For z-stack images, 0.125 µm steps in the z-direction were taken. The pinhole size was set to 499.69 mAU. Sections were irradiated with the following laser lines: 405, 488, 638. Each image was detected on 3 × spectral HyD S detector using HC PL APO CS2 63 ×/1.4 lens. The appropriate spectra were defined by the ImageCompass software (Leica Microsystems).

### Image analysis

All images were processed using FIJI software. Manders Overlap Coefficient (MOC) was calculated using Colocalization Threshold plug-in available in FIJI. The thresholds were defined using Costes automatic threshold searching [22]. To measure the LRP10-positive vesicle area in post-mortem brain tissue, we acquired 6–9 Z-stack images of the substantia nigra with a scan size of 4096 × 4096 and a pixel size of 0.946 µm. We only included glial cells residing in the SN based on the presence of LRP10-positive vesicles. All the glial cells were selected, irrespective of the presence of any pathological feature. We then performed automated batch analysis using FIJI batch processor with a self-generated macro-code on maximum projection regions of interests (ROIs) containing single cells expressing LRP10-positive vesicles. First, we subtracted background noise using the rolling ball algorithm with a radius of 10. Gaussian blur filter (*σ* = 2.0) was applied to all images. Vesicle segmentation was then performed using the auto threshold method developed by Otsu [64]. Images were converted to masks, and watershed was applied to segment adjacent structures. Segmented LRP10-positive vesicles were then analysed with the Analyze Particles plug-in. Very small particles with a size below 0.5 µm^2^ were discarded from the analysis. For quantitative characterisation of iPSC-derived progenitors, vmDAN, and astrocytes, fluorescence-based thresholding was applied for each cell-specific marker. Cells were considered positive for each marker when their fluorescent signal was above that threshold. To measure the percentages of LRP10 immunoreactivity in various types of Lewy pathology, we acquired Z-stack images and only selected neuromelanin-containing dopaminergic neurons of the SN. For the analysis, we considered neurons with morphologically diverse α-synuclein immunoreactive deposits within the neuronal soma. We identified diverse types of α-synuclein accumulations as described by Kuusisto et al. [44] and grouped the cells based on their shared α-synuclein immunostaining profile. For the analysis of LRP10 regional expression in the adult human brain tissue from non-demented subjects, we acquired Z-stack images and selected for astrocytes based on the expression of the astrocyte-specific marker S100β in the substantia nigra, temporal pole, and throughout the hippocampus. Fluorescence-based thresholding was applied for S100β. Cells were included in the analysis when their fluorescent signal for S100β was above that threshold. For 3D volume renderings, LAS X 3D (Leica Microsystems) module was used. For 3D surface renderings, Imaris Viewer (Oxford Instruments) was used.

### Statistical analysis

Statistical analyses were carried out using GraphPad Prism 8 (San Diego, USA). Outliers were identified using Grubbs’ (alpha = 0.05) or ROUT (*Q* = 1%) testing. One-way ANOVA with post hoc Tukey test or unpaired t test were applied for the analysis of in vitro experiments. Non-parametric methods were applied on non-normally distributed data. When comparing three or more groups, the Kruskal–Wallis test with Dunn’s multiple comparison post-test was used. *P* value of < 0.05 or lower reflected statistical significance.

## Results

### Characterisation of specificity of novel LRP10 antibodies

To determine endogenous LRP10 protein expression in cultured cells and human brain tissue, we assessed the specificity of LRP10 antibodies by Western blotting, immunoprecipitation, and immunocytochemistry. We generated LRP10 KO cells in two independent LRP10-expressing cell lines (HEK293T and HuTu 80) via CRISPR/Cas9 genome editing using a single guide RNA (sgRNA) that targets the first exon of the *LRP10* gene (Online Resource Fig. 1a) [10, 40].

**Fig. 1:**
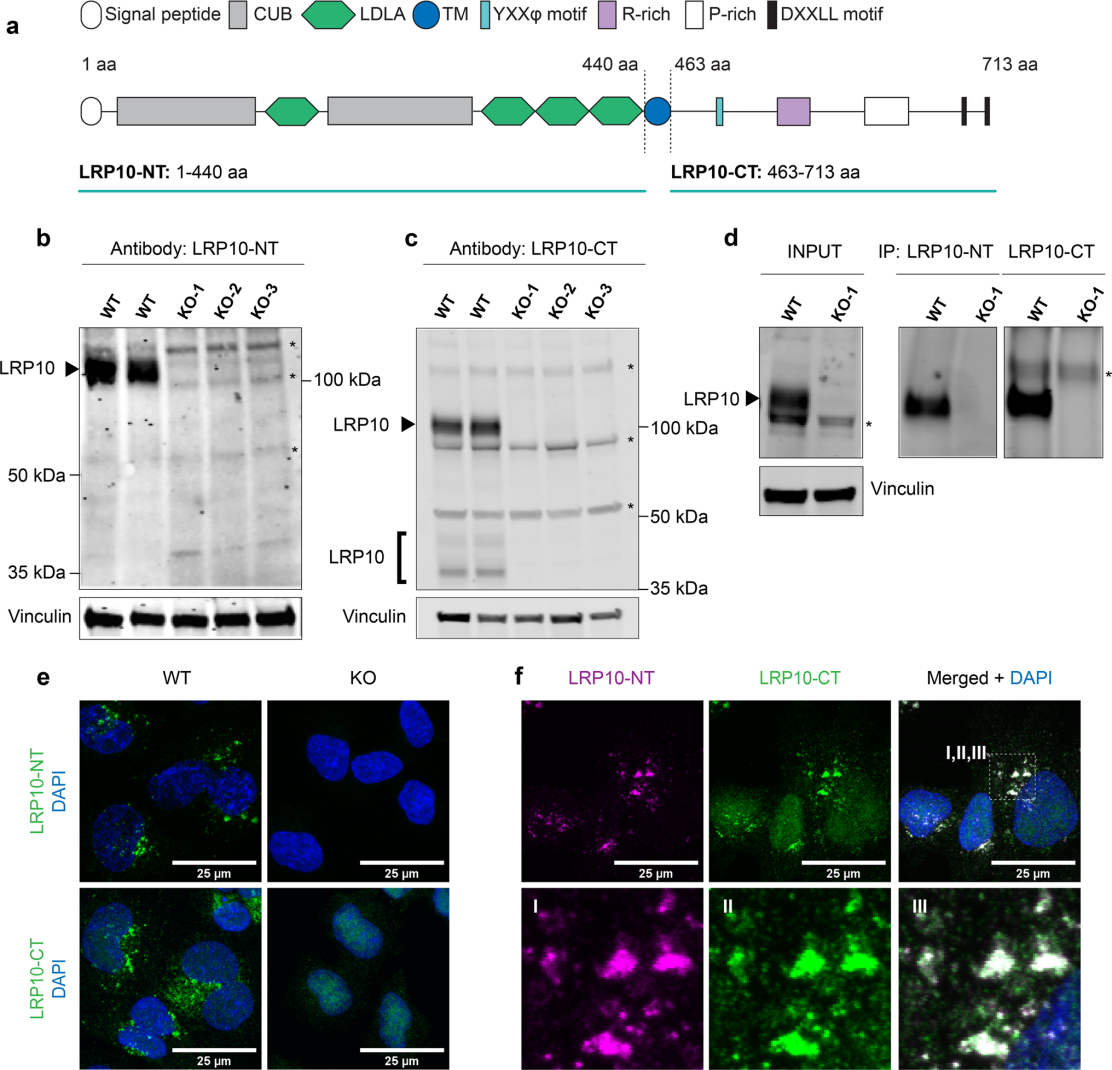
Validation of LRP10 antibodies. **a** LRP10 protein structure. LRP10 antibodies were raised against the extracellular/luminal domain (LRP10-NT: 1–440 aa) or the intracellular/cytosolic domain (LRP10-CT: 463–713 aa). CUB, complement C1R/C1S, urchin EGF, BMP1; LDLA, low-density lipoprotein receptor class A; TM, transmembrane domain; R-rich, arginine-rich domain; P-rich, proline-rich domain; YXXφ motif of a tyrosine plus two other amino acids followed by an amino acid with a large bulky hydrophobic side chain; aa, amino acid. Western blots showing endogenous LRP10 in LRP10 WT and KO lines (black arrows) using extracellular (**b**) or intracellular (**c**) LRP10 antibody for detection. Whole cell lysates were obtained from HEK293T clones. Vinculin was used as a reference. asterisk, non-specific binding. **d** Immunoprecipitation of endogenous LRP10 from LRP10 WT and KO lines generated in HEK293T using both LRP10 antibodies. Input LRP10 was detected using LRP10-CT antibody. Immunoprecipitation with LRP10-NT, detection with LRP10-CT; immunoprecipitation with LRP10-CT, detection with LRP10-NT. Asterisk, non-specific binding. **e** Representative images of LRP10 (green) in LRP10 WT and KO lines from HuTu 80 cells. **f** Co-localisation of extracellular (magenta) and intracellular (green) LRP10 antibody in HuTu 80 cells. For **e** and **f**: maximum intensity projections. Scale bars, 25 µm. Nuclei were counterstained with DAPI (blue)

We selected biallelically targeted clonal lines carrying homozygous or compound heterozygous frameshift mutations, predicted to result in a premature stop codon in *LRP10* (KO-1, KO-2, KO-3 in HEK293T or HuTu 80; Online Resource Fig. 1b, c) and examined them at the protein level. Seven commercially available antibodies raised against human LRP10 protein (Online Resource Table 4), as well as a novel polyclonal antibody recognizing the C-terminal domain, hereafter referred to as LRP10-CT (Thr463–Thr713, Fig. 1a), were assessed by Western blotting. Out of these in total eight antibodies, one commercial antibody (#13228-T16, Sino Biological) directed against the N-terminal LRP10 region, hereafter referred to as LRP10-NT (Met1-Lys440, Fig. 1a), as well as the LRP10-CT antibody showed a clear loss of LRP10 signal at the expected molecular weight of 100 kDa [18] in protein extracts derived from LRP10 KO cells generated in HEK293T cells (Fig. 1b, c) or Hutu 80 cells (Online Resource Fig. 1d, e). Interestingly, additional signals detected in the range of 35–50 kDa when using the LRP10-CT antibody (Fig. 1c) were absent in all LRP10 KO clones and not detected using the LRP10-NT antibody (Fig. 1b). These bands might be indicative of shorter LRP10 isoforms or posttranslational processing of the LRP10 protein. Next, we show that both LRP10-NT and LRP10-CT antibodies are able to specifically interact with endogenous LRP10 protein expressed in HEK293T cells under non-denaturing and non-reducing conditions demonstrated by immunoprecipitation of LRP10 protein at the expected LRP10 molecular weight that was absent in LRP10 KO HEK293T cells (Fig. 1d). Finally, both LRP10-NT and LRP10-CT antibodies were validated by immunofluorescence in wild-type (WT) HuTu 80 cells, showing a vesicle-like staining pattern, which was absent in HuTu 80 LRP10-KO cells (Fig. 1e). Additionally, immunofluorescence labelling using both LRP10 antibodies simultaneously showed vesicle-like staining pattern that fully co-localised (Fig. 1f). Taken together, these data validate the LRP10-NT and LRP10-CT antibodies for sensitive and specific detection of the endogenous LRP10 protein under native and denaturing conditions, establishing an important tool for further characterisation of LRP10 expression in cultured cells and in the human brain.

### LRP10 expression in adult human brain

We first investigated the LRP10 cellular and regional distribution in the normal adult human brain.

**Fig. 2:**
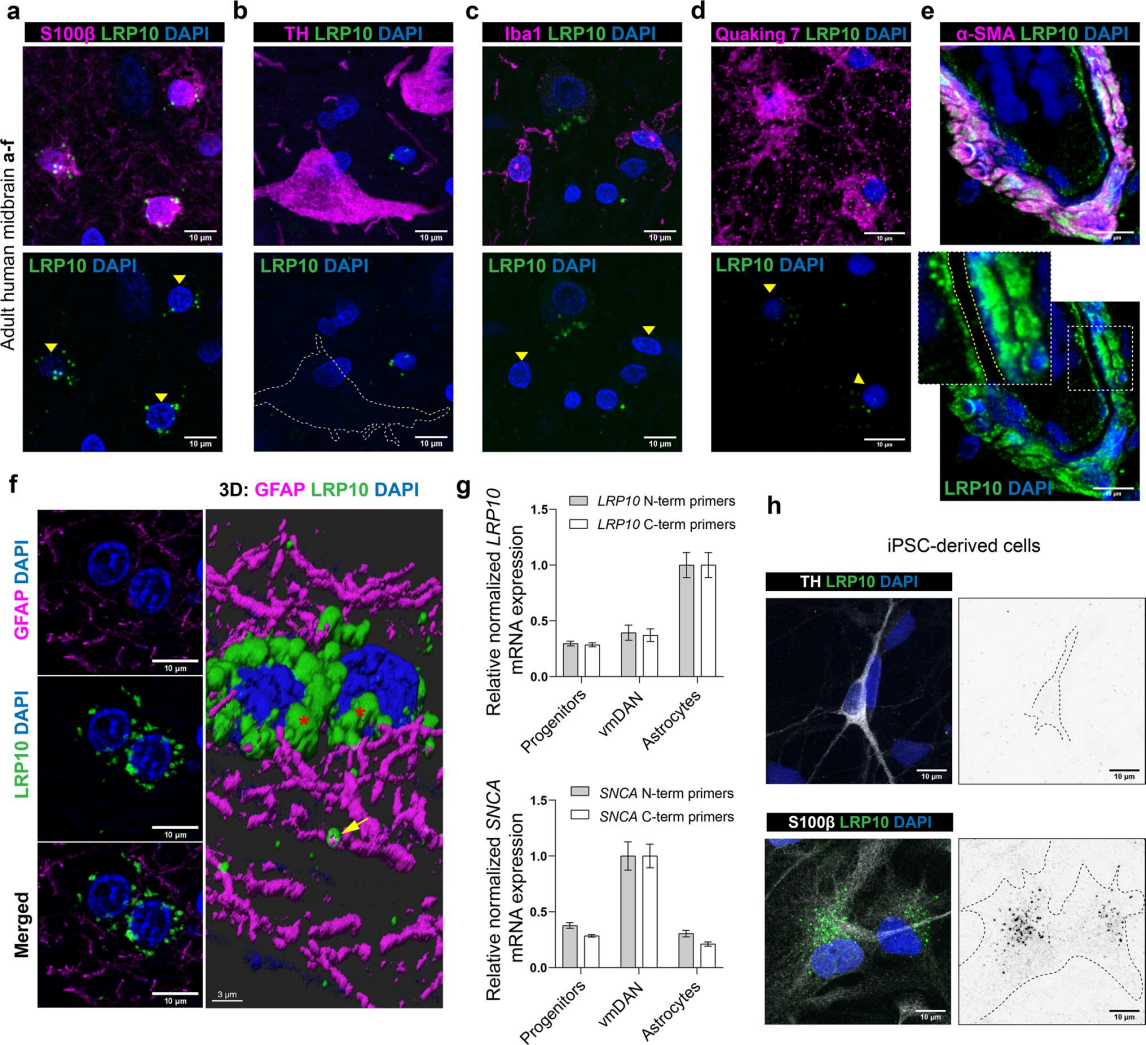
LRP10 is highly expressed in astrocytes and neurovasculature. **a–e** Representative images of LRP10 (green) protein expression at the cellular level in adult human midbrain. **a** Astrocytes were stained with S100β (magenta). **b** Dopaminergic neurons were stained with TH (magenta). **c** Microglia were stained with Iba1 (magenta). **d** Oligodendrocytes were stained with Quaking 7 (magenta), an RNA-binding protein that is highly specific for myelinating oligodendrocytes in the CNS. **e** Vascular smooth muscle cells were stained with α-SMA (magenta). Endothelial basement membrane is indicated between yellow, dashed lines. Yellow arrows indicate cells positive for either S100β, Iba1, or Quaking 7. **f** 3D surface rendering of LRP10 in astrocyte processes (GFAP, magenta) in adult human midbrain. LRP10 vesicle cluster around perinuclear region is indicated with red asterisks. Yellow arrows indicate partial co-localisation between LRP10 and GFAP. **g** RT-qPCR analysis of *LRP10* and *SNCA* genes in human iPSC-derived progenitors, vmDAN, and astrocytes from a control line. Data represent relative normalized mRNA expression using primers designed against N-term and C-term of *LRP10* or *SNCA* cDNA. *CLK2*, *COPS5*, *RNF10* were used as reference genes. Error bars represent ± SEM (*N* = 3 biological replicates). **h** Representative images of LRP10 protein expression in human iPSC-derived dopaminergic neurons (TH) or astrocytes (S100β). Representative images from a minimum of three independent differentiations. **a–f** Representative images from a minimum of three brain sections derived from three non-demented individuals. All stainings were performed with LRP10-NT antibody. Nuclei were counterstained with DAPI (blue). Maximum intensity projections. Scale bars, 10 µm. vmDAN, ventral midbrain dopaminergic neurons

By multi-label immunohistochemistry and confocal microscopy on FFPE human midbrain sections, using the LRP10-NT antibody, we detected vesicular LRP10 protein labelling primarily in S100 calcium-binding β (S100β) positive, mature astrocytes (Fig. 2a). The majority of LRP10-immunoreactive vesicles clustered around the perinuclear region, and only few co-localised within astrocyte processes labelled with glial fibrillary acidic protein (GFAP, Fig. 2f). Interestingly, LRP10 immunoreactivity was not detectable in dopaminergic neurons of the substantia nigra pars compacta (SNpc) (Fig. 2b) or in Iba1-positive microglia (Fig. 2c). Positive, weak LRP10 vesicular labelling was observed in Quaking 7-positive oligodendrocytes (Fig. 2d). Furthermore, we detected strong, diffuse LRP10 immunoreactivity in α-SMA-positive vascular smooth muscle cells, as well as in cells lining the inner vessel walls, most likely representing endothelial cells separated from the vascular smooth muscle cells by LRP10-negative endothelial basement membrane in the midbrain neurovasculature (Fig. 2e). This pattern of expression is in line with single-cell RNA sequencing from mouse neurovasculature [35, 84], revealing high expression of LRP10 in pericytes, smooth muscle cells, vascular fibroblast-like cells, endothelial cells, and astrocytes (Online Resource Fig. 3). Next, we analysed the astrocytic (S100β-positive) expression of LRP10 in different brain regions, including midbrain (SNpc), hippocampus, and temporal cortex. Interestingly, LRP10 protein levels calculated as amount of LRP10-immunoreactive vesicles per S100β-positive astrocyte were significantly higher in SNpc when compared to hippocampus or temporal cortex (Online Resource Fig. 2c, d; midbrain vs. temporal cortex, *p* < 0.001; midbrain vs. hippocampus, *p* < 0.001). This regional differential LRP10 protein expression is in line with the *LRP10* RNA microarray data obtained from six adult control brain donors of African American (2), Hispanic (1), and Caucasian (3) origins (http://www.human.brain-map/microarray/search, Allen Brain Atlas). The highest relative *LRP10* gene expression was found in brainstem nuclei, including midbrain, whereas low expression was detected in cortical regions, including hippocampus and temporal cortex (Online Resource Fig. 4a). These expression patterns were consistent across three LRP10-specific probes. To further investigate the striking observation that LRP10 expression is high in brain astrocytes, but low in TH-positive dopaminergic neurons, we analysed LRP10 protein expression in previously established in vitro models generating human iPSC-derived astrocytes and ventral midbrain dopaminergic neurons (vmDAN) from a healthy individual (Online Resource Fig. 2a) [71]. Robust expression of key markers of progenitor cells (99% Nestin, 84% SOX2), vmDAN (58% MAP2, 37% FOXA2), and astrocytes (51% AQP4, 60% SOX9) using immunocytochemistry confirmed the efficiency of the differentiation protocols used in this study (Online Resource Fig. 4b, c). Next, RNA expression analyses by RT-qPCR in these three different in vitro cell populations (progenitors, vmDAN, astrocytes) demonstrated higher *LRP10* expression in iPSC-derived astrocytes when compared to progenitor or vmDAN cultures (Fig. 2g), whereas *SNCA* expression levels were higher in neurons when compared to progenitor or astrocyte in vitro cultures (Fig. 2g). Finally, analyses of LRP10 protein expression by immunofluorescence using KO-validated LRP10-NT antibody demonstrates strong vesicular staining pattern in iPSC-derived astrocytes (Fig. 2h). This strong LRP10 immunoreactivity was not detected in vmDAN (Fig. 2h). Taken together, these data show that LRP10 expression is mainly restricted to non-neuronal cells (including astrocytes and neurovasculature resident cells) in vivo, and this differential astrocyte-enriched LRP10 expression is maintained in our in vitro model of human iPSC-derived vmDAN and astrocytes.

### Endogenous LRP10 mainly co-localises with early endosomes and the retromer complex

**Fig. 3:**
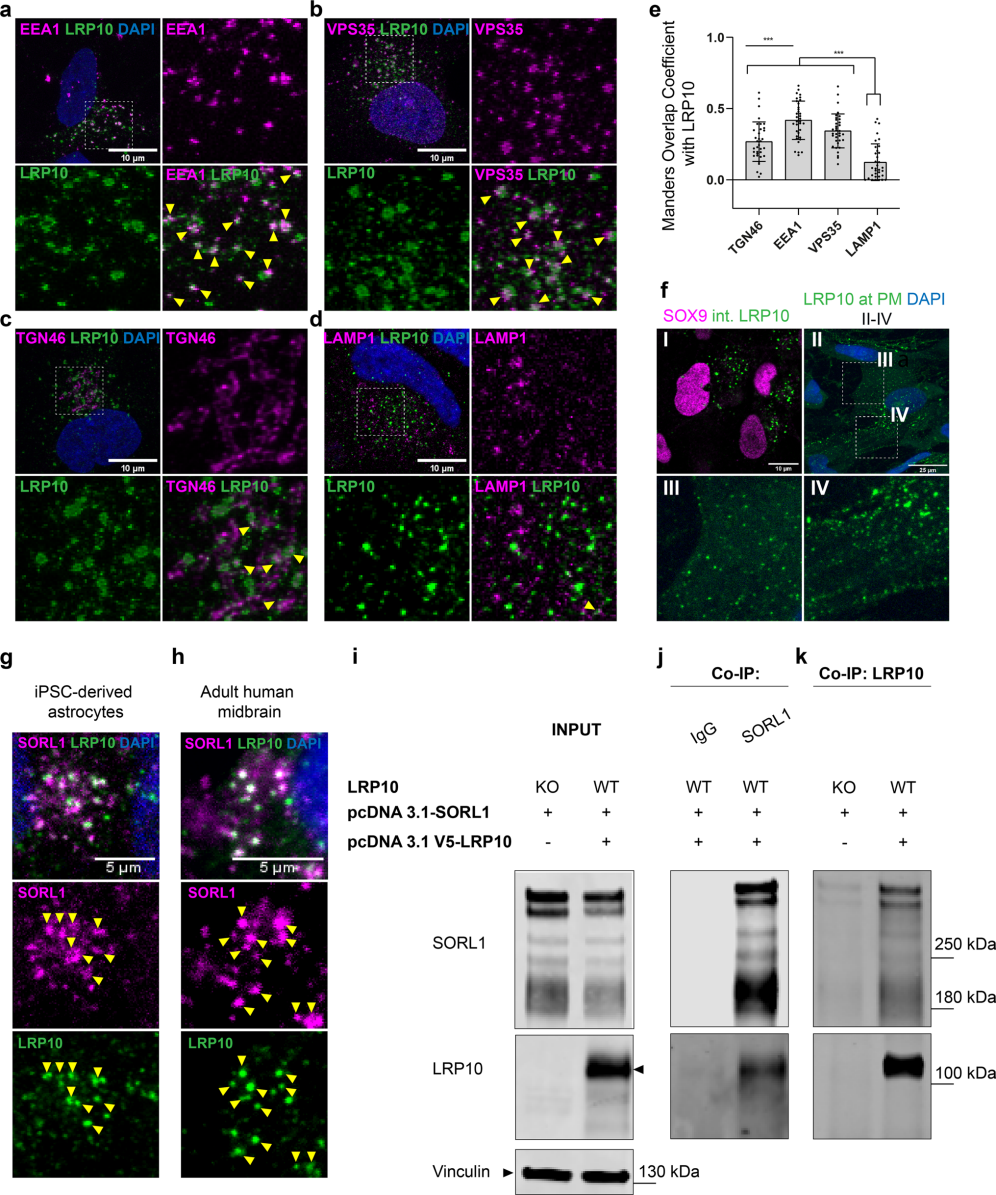
LRP10 is localised to vesicular structures and interacts with SORL1. **a–d** Deconvolved confocal images of LRP10 (green) co-localisation with various subcellular markers in 8 weeks old iPSC-derived astrocytes. **a** Early endosomes were detected using anti-EEA1 antibody (magenta). **b** The retromer complex was stained with anti-VPS35 antibody (magenta). **c** Trans-Golgi was stained with anti-TGN46 (magenta). **d** Lysosome-associated membrane protein 1 (LAMP-1) was used to detect lysosomal vesicles. **a–d** Zoomed images represent magnified views of boxed areas in the perinuclear region. Co-localisation with LRP10 is indicated by yellow arrowheads. Scale bars, 10 µm. **e** Quantification of LRP10 co-localisation with various subcellular markers. Thresholded Manders Overlap Coefficient is plotted on the y-axis. Per condition, 35 cells analysed. Error bars represent mean ± SD, one-way ANOVA with post hoc Tukey test; n.s., not significant; ***p < 0.001. **f** 11 weeks old human iPSC-derived astrocytes (SOX9+, magenta) were permeabilized and stained for intracellular LRP10 (green). For panels I and II: astrocytes were pre-incubated on ice to arrest endocytosis. Cell surface LRP10 molecules at the plasma membrane (PM) were labelled with LRP10-NT antibody. Scale bars, 10 µm. **g, h** Representative LRP10 (green) and SORL1 (magenta) co-localisation in iPSC-derived astrocytes (**g**) or in adult human midbrain (**h**). Images represent magnified views of boxed areas in the perinuclear region. Representative images from a minimum of three differentiations or three brain sections derived from three different non-demented individuals. Co-localisation LRP10 with SORL1 is indicated by yellow arrowheads. **i–k** HEK293T LRP10-KO or WT cells were transiently co-transfected with untagged full-length SORL1 only or together with N-terminally V5-tagged full-length LRP10. Total cell lysates (input) were subjected to immunoprecipitation with antibodies against SORL1 and LRP10. SORL1 antibody detected several bands (> 180 kDa) of overexpressed full-length SORL1 protein. Mouse IgG was used as a negative control. Immune complexes were resolved by SDS-PAGE, followed by Western blotting. **a–d, f–h** All stainings were performed with LRP10-NT antibody. Nuclei were counterstained with DAPI (blue). Maximum intensity projections

Our immunohistochemical experiments on human brain tissue showing vesicular LRP10 localisation (Fig. 2a, f) are in line with previous studies demonstrating that in various cell types, overexpressed and tagged LRP10 proteins mainly localise to the trans-Golgi network, endosomes, and the retromer complex [15, 18, 26, 70]. As the presence of protein tags and protein overexpression by itself can interfere with physiological protein localisation, we next set out to determine the subcellular localisation of endogenously expressed LRP10 protein using LRP10-NT antibody in combination with antibodies labelling organelle-specific proteins. In iPSC-derived astrocytes from a healthy individual, we detected strong co-localisation of LRP10 with the early endosomal marker EEA1 (Fig. 3a) and the retromer marker VPS35 (Fig. 3b). Furthermore, at the perinuclear region, LRP10 co-localised with the trans-Golgi network marker TGN46 (Fig. 3c). LRP10-labelled structures co-localised to a lesser extend with lysosome-associated membrane protein 1 (LAMP-1, Fig. 3d, e). To quantify the degree of overlap between LRP10 and various subcellular markers, the Manders Overlap Coefficient was calculated (Fig. 3e) showing the highest LRP10 co-localisation with early endosomes and the retromer (mean (SD); LRP10-EEA1: 0.42 (0.13); LRP10-VPS35: 0.34 (0.12); EEA1 vs. VPS35, *p* = 0.08) and moderate overlap with the trans-Golgi network [mean (SD); LRP10-TGN46: 0.27 (0.14)]. The degree of the LRP10 overlap with LAMP-1 was significantly lower when compared to EEA1, TGN46, or VPS35 (TGN46 vs. LAMP-1, *p* < 0.001; EEA1 vs. LAMP-1, *p* < 0.001; VPS35 vs. LAMP1, *p* < 0.001). To test whether endogenous LRP10 is localised to the PM, we incubated non-permeabilized cells at 4 °C with the LRP10-NT antibody, directed against the predicted extracellular/luminal domain of LRP10 [18], to specifically label PM-localised LRP10 proteins. Indeed, we detected diffuse as well as clustered LRP10-staining at the PM (Fig. 3f, panels II, III, and IV). In contrast, permeabilized astrocytes demonstrated high expression of nuclear localised astrocyte-enriched transcription factor SOX9 and LRP10-positive intracellular vesicles (Fig. 3f, panel I). These results are in line with previous overexpression studies and suggest that the normal trafficking routes of LRP10 involve shuttling from the Golgi apparatus to the PM, where after internalization LRP10 is transported to endosomes. From the endosomes, it can be either retrogradely transported through the retromer complex to the Golgi, or targeted via late endosomes towards lysosomes for its final degradation.

### LRP10 is a novel SORL1-interacting protein

The subcellular localisation of LRP10 described in this study, combined with the earlier proposed model for LRP10 as a sorting receptor for the amyloid precursor protein (APP) [18] and apolipoprotein E (APOE) internalization [19] show similarities with subcellular localisation and function of another member of the LDL-receptor family—SORL1. Interestingly, SORL1 has been genetically and functionally associated with AD [5, 74]. Because of these observed similarities and previously reported interactions between SORL1 and other members of the LDL-receptor family [79], we hypothesise that LRP10 and SORL1 proteins physically interact to form a receptor complex. To test this hypothesis, we first looked at their co-localisation in vitro and in vivo using immunocytochemistry and immunohistochemistry, respectively. Interestingly, both in iPSC-derived astrocytes (Fig. 3g) as well as in midbrain sections from control subjects (Fig. 3h), we detected high levels of overlap between SORL1 and LRP10 immunoreactivity, suggestive of a potential physical interaction between them. Next, to determine whether LRP10 and SORL1 are part of the same protein complex, we performed co-immunoprecipitation experiments. First, protein extracts from WT HEK293T cells expressing full-length V5-tagged LRP10 together with untagged full-length SORL1 (Fig. 3i, INPUT WT) or untagged full-length SORL1 alone (Fig. 3i, INPUT KO) were used as an input for co-immunoprecipitation experiments. Interestingly, using a mouse anti-SORL1 antibody, V5-LRP10 co-immunoprecipitated with untagged SORL1, but could not be detected using a nonimmune mouse immunoglobulin (IgG) as a negative control (Fig. 3j). Furthermore, the interaction between LRP10 and SORL1 was confirmed using HEK293T WT and LRP10 KO lines that were only transfected with the full-length untagged SORL1 plasmid (Fig. 3i, INPUT KO). Using the LRP10-NT antibody, SORL1 co-immunoprecipitated with V5-LRP10 in WT cells, whereas only low levels of SORL1 were co-immunoprecipitated in the LRP10 KO protein extracts (Fig. 3k). These results are in support of our hypothesis, that LRP10 and SORL1 are part of the same protein complex, providing new insights into their potential roles in shared pathogenic pathways in neurodegenerative disorders.

### Enlarged LRP10-positive vesicles are detected in brain of the LRP10 p.Arg235Cys variant carrier

**Fig. 4:**
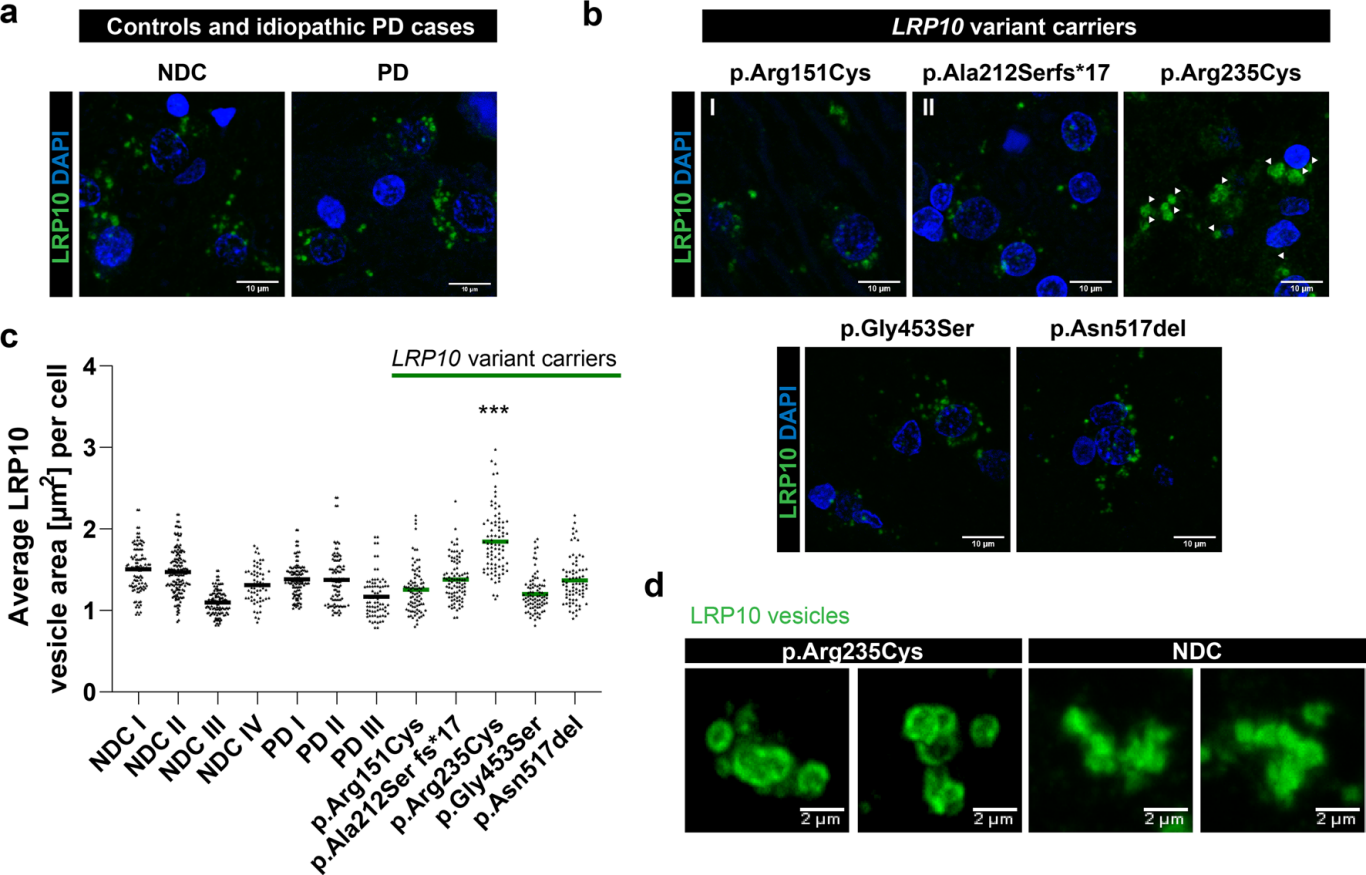
Enlarged LRP10-positive vesicles detected in brain of the LRP10 p.Arg235Cys variant carrier. **a, b** Representative images of LRP10 vesicle morphology from NDC (*N* = 4 individuals), PD (*N* = 3 individuals) (**a**), and five patients carrying *LRP10* pathogenic variants (**b**). Enlarged and clustered LRP10 vesicles were seen in the midbrain of the PDD patient carrying p.Arg235Cys variant (white arrowheads). Representative images from a minimum of three brain sections. Nuclei were counterstained with DAPI (blue). Maximum intensity projections. All stainings were performed with the LRP10-NT antibody. Scale bars, 10 µm. **c** Quantification of surface area (µm^2^) of LRP10-postive vesicles in controls, idiopathic PD and LRP10 variants carriers. Each value in the scatter dot plots represents the average vesicle size/cell. Kruskal–Wallis with Dunn multiple comparison post hoc; ***p < 0.001. *N* equals the number of cells analysed per brain specimen. *N*_NDC I_ = 108, *N*_NDC II_ = 157, *N*_NDC III_ = 112, *N*_NDC IV_ = 58, *N*_PD I_ = 112, *N*_PD II_ = 96, *N*_PD III_ = 80, *N*_p.Arg151Cys_ = 87, *N*_p.Ala212Ser fs*17_ = 89, *N*_p.Arg235Cys_ = 83, *N*_p.Gly453Ser_ = 85, *N*_p.Asn517del_ = 74. **d** High-resolution, re-scan confocal images of LRP10 vesicles reveal enlarged donut-like morphology in the PDD patient carrying p.Arg235Cys variant when compared to a non-demented control, scale bars 2 µm

To investigate the role of LRP10 in LBD pathogenesis, LRP10 expression was compared in autopsy-derived brain material from *LRP10* variant carrying patients, idiopathic PD cases, and age- and sex-matched control subjects (Online Resource Table 1). *LRP10* mRNA expression was unaltered in SNpc material from the idiopathic PD cohort (PD IV-IX, *N* = 6) when compared to non-demented controls (NDC VI-XI, *N* = 6) (Online Resource Fig. 5). Detailed clinical and pathological features of a total of five donors carrying rare, pathogenic variants in *LRP10* included in this study have been analysed elsewhere and are summarized in the materials and methods section [70, 86]. A severe burden of Lewy body pathology with the highest Braak α-synuclein stage was present in all these five LRP10 carriers in the brainstem, limbic, and neocortical areas [70, 86]. To determine whether LRP10 pathogenic variants affect LRP10 expression, we first analysed LRP10 staining intensity and area in midbrain sections derived from three different cohorts: non-demented controls (NDC, *N* = 4), idiopathic PD cases (*N* = 3), and *LRP10* variant carriers (patients I–V (*N* = 5); Online Resource Table 1) by immunohistochemistry using the LRP10-NT antibody. LRP10-positive vesicles were observed in a subset of SNpc cells from NDC, PD, and *LRP10* variant carriers (Fig. 4a, b). Although no apparent differences in size and staining intensity in LRP10-positive vesicles were observed between LRP10-expressing cells from NDC, PD and the majority of *LRP10* variant carriers, we observed significantly larger and clustered LRP10-positive vesicles in the brainstem tissue from the LRP10 p.Arg235Cys variant carrier (patient III, Fig. 4c; NDC I/II/III/IV vs. p.Arg235Cys, *p* < 0.001; PD I/II/III vs. p.Arg235Cys, *p* < 0.001; p.Arg151Cys/p.Ala212Ser fs*17/p.Gly453Ser/p.Asn517del vs. p.Arg235Cys, *p* < 0.001). Further analysis of the LRP10 vesicles in patient III by re-scan confocal microscopy [23] revealed donut-shaped vesicles, with a large lumen (Fig. 4d), reminiscent of enlarged and damaged lysosomes [13, 28].

### LRP10 is present in the core of Lewy bodies

**Fig. 5:**
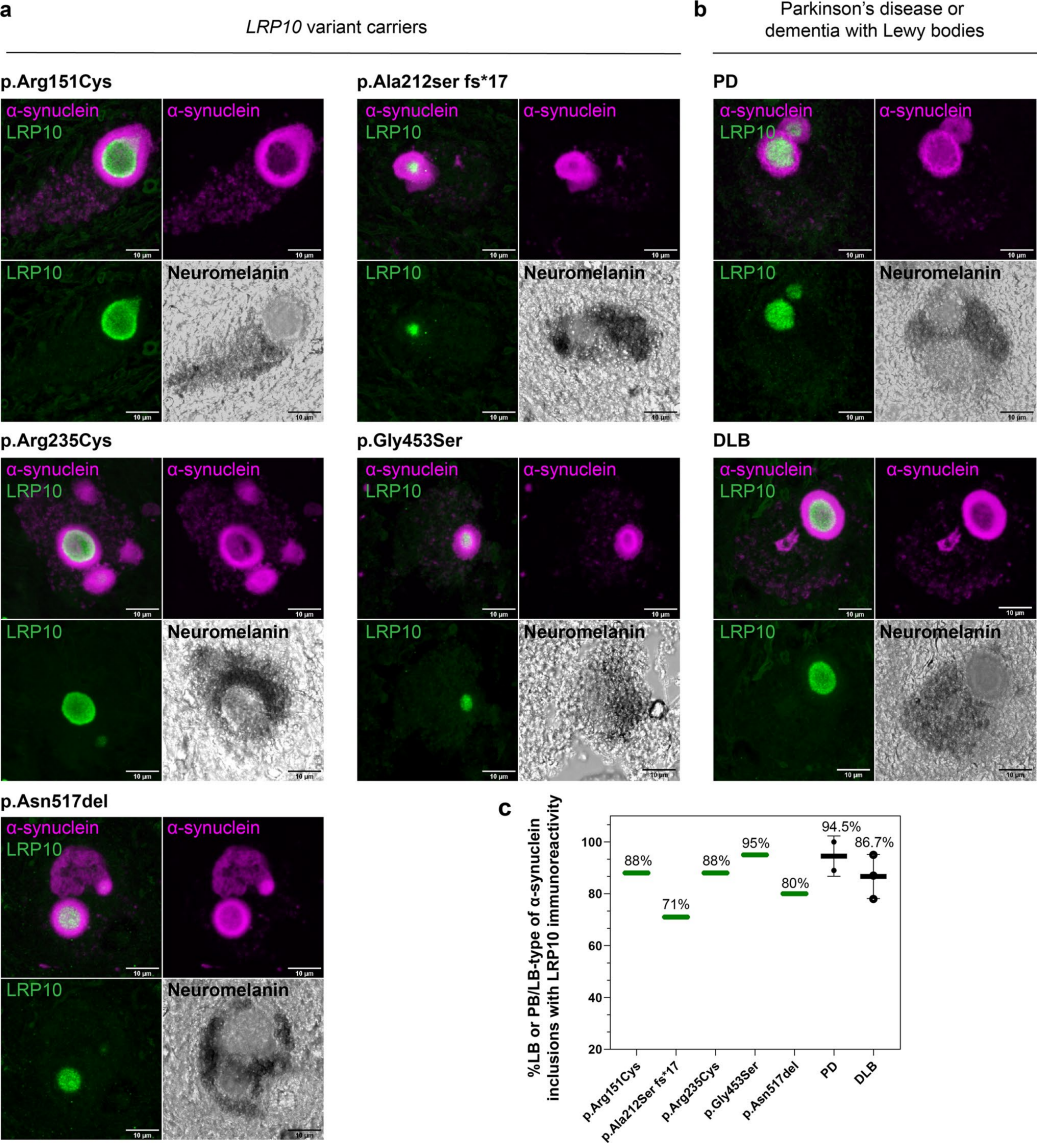
LRP10 immunoreactivity in the core of nigral Lewy bodies. **a, b** Double labelling of α-synuclein (SYN-1, magenta) with LRP10 (green) antibody recognizing the intracellular domain. LRP10 immunoreactivity is present in the core of a LB in neuromelanin-positive dopaminergic neurons of SNpc (bright-field images). Representative confocal images of nigral LBs in patients carrying LRP10 mutations (**a**), or idiopathic PD and DLB (**b**). Maximum intensity projections. Scale bars, 10 µm. **c** Quantification of percentages of LRP10 immunoreactivity in more mature PB/LB-type or LB-type of α-synuclein inclusions. All inclusions analysed were found in neuromelanin-containing dopaminergic neurons of substantia nigra. *N* = 20 LB per individual; *N*_DLB_ = 3 individuals (60 LB); *N*_PD_ = 2 individuals (40 LB). Error bars represent mean ± SD. LB, Lewy body

To further determine the role of LRP10 in Lewy pathology, we analysed LRP10 protein expression and localisation in relationship to LB-containing neurons in brain specimens obtained from the *LRP10* variant carriers, idiopathic PD and DLB patients. The ring-shaped appearance of strong peripheral α-synuclein immunoreactivity and a weakly stained core typical of brainstem-type, mature LBs were revealed in neuromelanin-positive cells of SNpc sections from all analysed cases using a validated immunohistochemical staining protocol for α-synuclein (SYN-1) antibody [66, 82]. Strikingly, co-staining of LRP10 and α-synuclein demonstrated the presence of LRP10 immunoreactivity in the core of brainstem-type, mature LBs in SNpc from all *LRP10* variant carriers as well as idiopathic PD and DLB patients (Fig. 5a, b). Quantification of LRP10-positive LB in neuromelanin-containing neurons demonstrated a high percentage of LRP10-positive LBs (71–91%, mean = 86% LRP10 + LB (*N* = 200), Fig. 5c). In temporal cortex and medial frontal gyrus (Online Resource Fig. 6) from a total of seven brain donors, we only found few cortical LBs with weaker LRP10 immunoreactivity in the form of sparse vesicles (Online Resource Fig. 6c) or diffuse staining in the core of a targetoid cortical LB (Online Resource Fig. 6d). Taken together, these data identify LRP10 as a novel LB-resident protein mainly found in brainstem-type Lewy pathology.

**Fig. 6:**
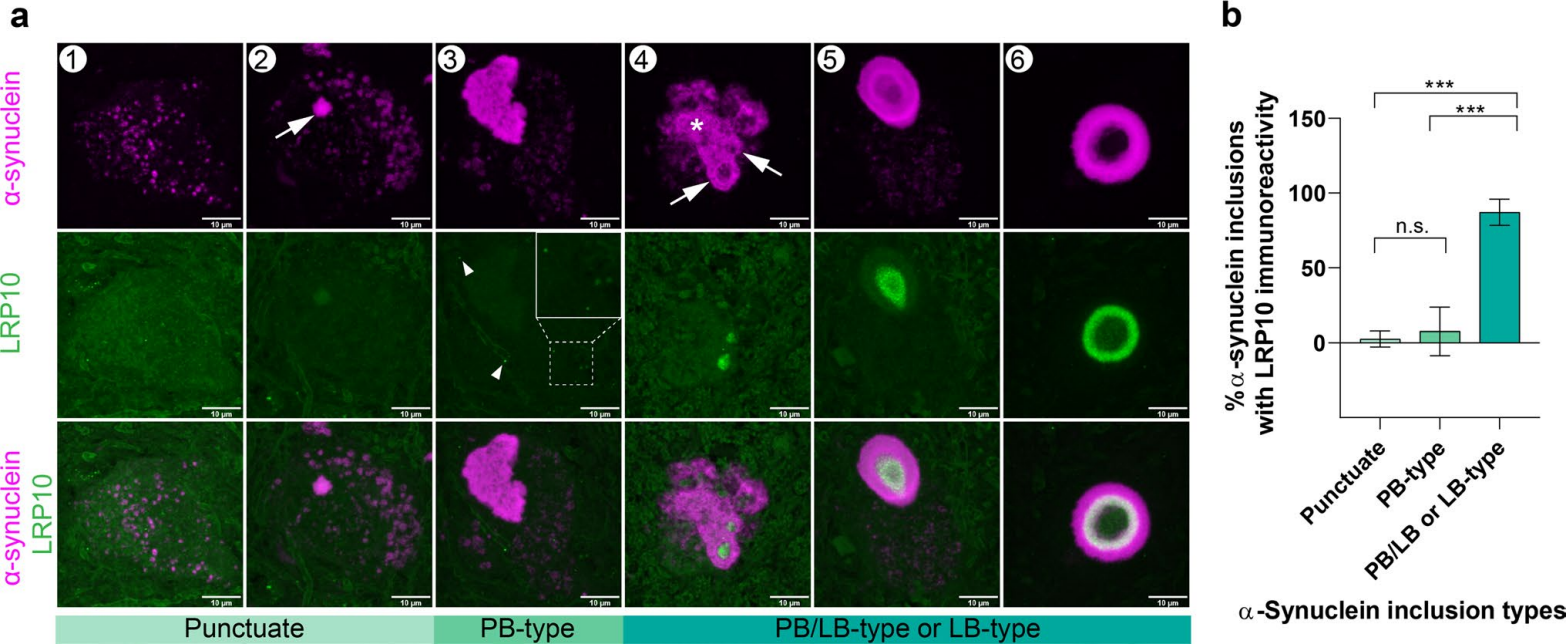
Incorporation of LRP10 into α-synuclein inclusions during LB formation. **a.** Representative confocal images of morphological spectrum of α-synuclein (SYN-1, magenta) inclusion formation (steps 1–6). LRP10 (green) incorporation is present in more mature PB/LB or LB-type of α-synuclein inclusions. 1. Intracytoplasmic, punctuate α-synuclein. 2. Intracytoplasmic, punctuate α-synuclein with an aggregation centre (arrow)**.** 3. PB-type: less compact, unstructured α-synuclein inclusion. Arrow heads and zoomed area indicate LRP10 vesicles. 4. Intracytoplasmic α-synuclein network containing PB (asterisk) and more compact LB-type inclusions (arrow). 5. Intracytoplasmic α-synuclein network with LB-type inclusion. 6. Mature LB. Maximum intensity projections. Scale bars, 10 µm. Representative images from a total of 247 nigral α-synuclein inclusions imaged. All inclusions analyzed were found in neuromelanin-containing dopaminergic neurons of substantia nigra. **b** Percentages of LRP10 immunoreactivity in different patterns of α-synuclein inclusions. *N*_total_ = 247; *N*_punctuate_ = 51; *N*_PB-type_ = 44; *N*_PB/LB-type or LB-type_ = 152. Kruskal–Wallis with Dunn multiple comparisons test; n.s., not significant; ***p < 0.001. LB, Lewy body. PB, pale body

### LRP10 protein is detected at late LB maturation stages

Different LB maturation stages associated with LB pathology in PD and DLB have been described to reflect LB morphogenesis [44]. We, therefore, characterised at what LB maturation stage LRP10 protein is incorporated into nigral LBs using co-staining of LRP10 and α-synuclein. First, we identified 6 LB maturation stages (Fig. 6a) based on α-synuclein staining patterns, including cytoplasmic, punctuate pattern (1), punctuate pattern with a starting α-synuclein aggregation centre (2), large, proteinaceous accumulations, termed pale bodies (PB) (3), a combination of irregular accumulation with a progressive condensation into a compact LB-type of inclusion (4), cytoplasmic, punctuate α-synuclein pattern with a defined and compact LB (5), mature LB (6) (Fig. 6a, panels 1–6). LRP10 was not detected in neuromelanin-containing dopaminergic neurons containing stage 1 and 2 types of α-synuclein-positive inclusions (Fig. 6a, panels 1 and 2). However, sparse LRP10-positive vesicles could be detected in neurons containing PB-type inclusions (Fig. 6a, panel 3). Interestingly, as the condensation of α-synuclein aggregates progressed to stage 4, LRP10 immunoreactivity was observed in the centre of immature inclusions (Fig. 6a, panel 4). At the final two maturation stages, granular LRP10 staining was detected in the LB core of α-synuclein inclusions in neuromelanin-positive neurons (Fig. 6a, panels 5 and 6). Additionally, we used super-resolution microscopy to further characterise the presence of LRP10 protein in the core of nigral LBs. With this approach, we observed numerous LRP10-positive vesicular structures in the interior of these mature α-synuclein inclusions (Online Resource Video 1 and 2). Quantification of LRP10 immunoreactivity in α-synuclein inclusions revealed that LRP10 is mainly present at the last three stages of LB morphogenesis (Fig. 6b; *N*_total_ = 247, *N*_punctuate_ = 51, *N*_PB-type_ = 44, *N*_PB/LB-type or LB-type_ = 152, *p* < 0.001). No difference in the maturation pattern of LRP10 incorporation into nigral LB was observed when comparing LB localisation of LRP10 in idiopathic PD (*N* = 3) and DLB (*N* = 3), and LRP10 pathogenic variant carriers (*N* = 5). Finally, recent reports demonstrated concentric laminar structure of LBs as identified by staining using α-synuclein antibodies targeting different α-synuclein domains [60]. Moreover, patterns of vesicular structures and dysmorphic organelles have been detected in Lewy pathology [76]. We performed multi-label microscopy including markers for lysosomal proteins, LAMP1 and LAMP2, and an early endocytic marker RAB5A to determine in more detail LRP10 protein localisation within the LBs with respect to organelle markers. Interestingly, these markers showed a distinct pattern characterised by concentric laminations in mature LBs, with LRP10 being consistently localised in the LB core, furthest from the periphery (Online Resource Fig. 7). These distinct patterns of incorporation and localisation of LRP10-positive vesicles in the core of mature LBs suggest an organized processing and mechanistic links between vesicle trafficking and α-synuclein inclusion formation in LBD.

## Discussion

A growing amount of genetic evidence supports the involvement of *LRP10* gene variants in the development of inherited forms of PD, PD with dementia, and DLB [20, 70, 86, 88]. In the present study, we provide novel insights into the expression and potential function of LRP10 in vitro and human brains in physiological and pathological conditions. Using novel, KO-validated and biochemically characterised LRP10 antibodies, we demonstrate the molecular characteristics, as well as brain regional, cellular, and subcellular localisation patterns of endogenously expressed LRP10 protein in iPSCs and human brain tissue.

LRP10 is a single-pass transmembrane protein and a distant relative of the LDL-receptor family with a predicted molecular weight of 76 kDa [67]. Yet, a higher molecular weight band of approximately 100 kDa is consistently detected by Western blotting using both LRP10-NT and LRP10-CT antibodies (Fig. 1b, c). This is likely caused by posttranslational modifications, as LRP10 has several predicted N-glycosylation sites and a phosphorylation site (http://uniprot.org). Interestingly, only the LRP10-CT antibody detects additional proteins in the range of 35–50 kDa, which are not detected in LRP10 KO cell lines. These fragments suggest the presence of other LRP10 isoforms possibly lacking the epitope detected by the LRP10-NT antibody. Two other protein isoforms of human LRP10 have been reported in addition to its full-length transcript (https://www.proteinatlas.org/ENSG00000197324-LRP10). However, these isoforms lack a large portion of the C-terminal region of LRP10, which is detected by the LRP10-CT antibody, and the molecular weight of these isoforms would be larger than the fragments detected here by Western blotting, making it unlikely that the detected 35–50 kDa products correspond to the above-mentioned LRP10 isoforms. Interestingly, it has been shown that some members of the LDL-receptor family can be processed at the cell surface by metalloproteinases (e.g. ADAM10) or secretases (BACE1 or γ-secretase), producing different protein fragments with various physiological functions [6, 67]. Further studies are needed to understand whether LRP10 undergoes proteolytic cleavage, and if so, what are the involved proteases and the physiological functions of LRP10 processed fragments.

Next, our expression analysis demonstrates that LRP10 is detected in non-neuronal cells with the highest expression levels in astrocytes and neurovascular unit in the adult human brain (Fig. 2 and Online Resource Fig. 3). Strikingly, we were unable to detect LRP10 signal in unaffected dopaminergic neurons in control subjects. This differential expression was also observed in iPSC-derived vmDAN and astrocytes, lacking or highly expressing LRP10, respectively. These findings are in line with publicly available online transcriptomic bulk and single-cell RNA expression databases, showing the highest LRP10 expression in mature astrocytes and lack of its expression in neurons [9, 45, 93]. We also provide evidence of differential astrocytic LRP10 protein expression in different brain regions, showing higher levels in the brainstem when compared to the hippocampus or temporal cortex in physiological conditions. This is also in line with expression data in existing databases, demonstrating that *LRP10* mRNA is highly expressed in the midbrain and diencephalon, as well as the dorsal motor nucleus of the vagus nerve, raphe nuclei, and coeruleus–subcoeruleus complex. Interestingly, these brain regions are proposed to be involved in the early stages of PD [16], suggesting therefore, a potential involvement of LRP10 in the selective regional vulnerability during the disease progression.

The specific LRP10 expression patterns suggest a prominent role of non-neuronal LRP10-mediated pathways in LBD pathophysiology where various disease mechanisms could be involved. First, astrocytes are essential in providing a trophic environment for neurons by secreting survival factors [8, 51], regulating synaptic functions [4], controlling brain homeostasis [78], and maintaining the blood–brain barrier (BBB) [2]. Disturbances of these neuron survival-promoting functions by astrocytes have been described in PD [51, 58]. Alternatively, astrocytes can also become reactive via a variety of challenges, such as activation by microglia, ageing, and neurodegeneration, generating an A1 astrocyte population with neurotoxic properties [21, 50]. These A1 astrocytes have been shown to be increased in major neurodegenerative disorders, including PD [50]. Additionally, a drug-mediated blockage of A1 astrocyte activation by pathological α-synuclein has been suggested to be neuroprotective in PD [91]. Interestingly, LRP10 levels appear to be increased in A1 reactive astrocytes when compared to naïve (A0) astrocytes (https://nyscfseq.appspot.com/*)*. Furthermore, astrocytes have been shown to regulate the cell-to-cell transfer of α-synuclein through various mechanisms [47, 73, 90]. Astrocytes can internalise α-synuclein, which can subsequently be processed via the autophagy–lysosome pathway, highlighting the importance of astrocytic α-synuclein removal and degradation in LBD [25, 55, 80]. Additionally, recent studies provide accumulating evidence that other PD genes play important roles in astrocyte biology, and their dysfunction might lead to neuronal α-synuclein aggregation and neuronal loss by non-cell autonomous mechanisms [14]. Based on our findings, we speculate that LRP10 could be involved in internalisation, degradation or cell-to-cell transport of α-synuclein by astrocytes.

In addition to astrocytes, we show high LRP10 expression in the neurovasculature, which plays an essential role in the clearance of proteins prone to aggregation, e.g. amyloid beta (Aβ) and possibly α-synuclein [11, 89]. Interestingly, other lipoprotein receptors (e.g. LRP1) have been shown to play important roles in the clearance of Aβ across the BBB [24]. Moreover, it has been suggested that LRP10 is an APP sorting receptor and reduced LRP10 levels lead to an increase in Aβ production [18]. Hence, similar to LRP1, LRP10 might play a role in the processing and clearance of aggregation-prone proteins at the gliovascular interface. We detect high LRP10 expression at various cellular components of the BBB, including endothelial and smooth muscle cell layers of the neurovasculature. Functional roles for LRP10 at the neurovasculature and BBB and whether LRP10 variants contribute to neurovascular or BBB dysfunction in LBD remain to be experimentally tested.

We show that endogenously expressed LRP10 localises to various subcellular compartments, including trans-Golgi, endosomes, plasma membrane, retromer, and, to a lesser extent, lysosomes. These data point towards deregulated intracellular trafficking pathways in PD and DLB patients harbouring loss-of-function variants in the *LRP10* gene. Recent advanced microscopic and genetic studies have brought forward a central role for impaired vesicle trafficking in PD’s pathogenesis [29, 76]. Interestingly, other PD-related genes such as *LRRK2*, *VPS35*, and *GBA* play essential roles in astrocytes, and it has been shown that variants in these genes lead to impairments in intracellular vesicle trafficking, dopaminergic neuron degeneration, and accumulation of toxic α-synuclein species in surviving neurons and glia [3]. Whether the non-neuronal expression of *LRP10* disease-associated variants leads to changes in intracellular vesicle trafficking needs to be further investigated.

Based on the data from this study and previous reports, LRP10 expression and its association with neurodegenerative diseases demonstrate a high degree of overlap with SORL1, an established causative gene and risk factor in AD [38, 72]. First, both LRP10 and SORL1 are transmembrane proteins that function at the plasma membrane and in intracellular vesicles, where they both mediate trafficking of APP to protect it from amyloidogenic processing [5, 18]. Next, both LRP10 and SORL1 need the retromer for the endosomal sorting, and when this interaction is abolished, LRP10 and SORL1 do not reach the plasma membrane [30, 81]. Here, we show strong co-localisation and protein–protein interaction between LRP10 and SORL1, providing evidence for converging functional pathways. How SORL1 and LRP10 functionally interact and how this interaction is affected by disease-associated LRP10 and SORL1 in LBD and AD variants remain to be determined. Our finding provides novel and exciting evidence of intriguing links between these proteins and the role of transmembrane protein sorting mechanisms in the development of neurodegenerative disorders, including PD, DLB, and AD.

To better understand the role of LRP10 expression in the diseased brain, we analysed LRP10 protein expression in brain specimens from five patients carrying heterozygous variants in the *LRP10* gene (p.Arg151Cys, p.Ala212Ser fs*17, p.Arg235Cys, p.Gly453Ser, p.Asn517del) with varying phenotypes from a typical late-onset PD to PD with dementia, or DLB. In most *LRP10* variant carriers, we did not observe significant changes in LRP10 vesicular staining patterns or area in comparison to NDC or idiopathic PD brain samples. However, in the SNpc material from the p.Arg235Cys variant carrier, donut-shaped LRP10-positive vesicles with significantly enlarged lumen were found. While we were unable to determine the precise identity of these enlarged vesicular structures, intriguingly, LRRK2 has been shown to regulate the size and number of lysosomes in primary mouse astrocytes, and mutant LRRK2 is recruited onto enlarged lysosomes [28, 37]. Furthermore, loss of SORL1 leads to enlarged early endosomes [42]. Both LRRK2 and SORL1 phenotypes are similar to the LRP10-positive structures observed in the p.Arg235Cys variant carrier in this study, pointing towards a potential impairment of endo-lysosomal pathways in patients with this variant. However, these results are based on a single brain, and they need to be interpreted with caution. Therefore, analyses of other carriers are warranted.

Comparing post-mortem tissues from controls and idiopathic PD, we show no significant differences in *LRP10* mRNA levels in substantia nigra. Additionally, LRP10-positive intracellular vesicle number and morphology were unaltered in the idiopathic PD cohort when compared to NDC. Other studies reporting on LRP10 expression levels in other neurodegenerative diseases show inconsistent results. A previous study found no significant difference between *LRP10* mRNA levels in patients with AD and controls but significantly reduced LRP10 protein expression was detected in the frontal cortex and hippocampus of AD brains by Western blotting [18]. It should be noted that the LRP10 antibody used in that study did not pass our antibody validation test for detecting the endogenous LRP10 protein. Additionally, LRP10 signals in AD and control samples were normalized to the neuronal Tuj1 protein, whereas based on our findings, normalization with general or astrocytic markers would have been more appropriate. Furthermore, a systematic multi-cohort transcriptomic analysis of post-mortem brain tissue from AD, Huntington’s disease (HD), PD, and ALS detected increased levels of *LRP10* in astrocytes in these neurodegenerative disorders [49]. Finally, a recent transcriptomic study on extensive AD cohorts demonstrated that LRP10 acts as a key driver of specific molecular subtypes of AD, with its expression being, either upregulated or downregulated in astrocytes, endothelial cells and microglia, depending on the AD subtype analysed [62]. Based on these studies and our findings, additional work to completely characterise LRP10 protein expression in brains of LBD and other neurodegenerative diseases is warranted.

Recent studies highlighted the presence of vesicles, fragmented organellar components, and lipids in LBs [57, 60, 76]. Moreover, various proteins implicated in the development of PD and related LB disorders, e.g. LRRK2 and GBA, are present in α-synuclein inclusions [32, 33]. Here, we detected LRP10-positive vesicle clustering in the core of brainstem-type mature LBs from idiopathic PD and DLB, as well as *LRP10* variant carriers. This observation suggests that LRP10-positive vesicles are either passively trapped in the core during LB maturation or could potentially play a more active role in LB growth. Furthermore, as the LRP10-specific antibodies used in our study cannot distinguish between WT and mutant protein, it remains unclear whether both proteins are localised to mature LBs. Interestingly, we found that LRP10 was barely detectable in cortical LBs, in keeping with the argument that they represent a less mature state when compared to brainstem type of LBs. Our data are also in line with another study, where LRP10 was not listed among 300 identified proteins in cortical LBs [48]. Furthermore, additional research is required to fully characterise LRP10 localisation in α-synuclein inclusions in all major synucleinopathies. In particular, it would be interesting to determine LRP10 localisation in α-synuclein-positive glial cytoplasmic inclusions (GCIs) in multiple system atrophy (MSA) and to understand whether LRP10 accumulation is only specific to mature brainstem-type LBs in PD and DLB.

We detected distinct LRP10-positive vesicle arrangements associated with various α-synuclein aggregation patterns in neuromelanin-containing dopaminergic neurons, reflecting previously described LB maturation stages [44]. Therefore, we speculate that LRP10 vesicles are undetectable in healthy dopaminergic neurons or at the initial stages of LB formation due to a very low abundance of LRP10 in neuronal cells and a still properly functioning degradation system. Subsequently, as the protein degradation systems (e.g. ubiquitin–proteasome and autophagy–lysosomal pathway) collapse and alignment of aggregated α-synuclein proceeds, LB morphogenesis progresses and LRP10 becomes apparent in the neuronal soma. At the final stages of LB remodelling and condensation, LRP10-positive vesicles aggregate in the core of LB together with other organellar components and lipids. This model of LRP10 incorporation into brainstem-type LBs requires either neuronal expressed LRP10 protein or LRP10 secretion by non-neuronal cells and its internalisation by neurons from the interstitial space towards LB morphogenesis. First, we speculate that if LB localised LRP10-positive vesicles have a neuronal origin, they could become apparent only during pathological conditions either via increased LRP10 expression or via impaired degradative processes in neurons during disease. We do not have evidence that LRP10 expression increases in PD patients (Online Resource Fig. 5). However, evidence for impaired degradative function has been reported. For instance, α-synuclein pathology and reduced GBA function have been linked to impairment of the autophagy–lysosomal pathway [7, 56, 75]. Interestingly, previous work demonstrated LRP10 degradation via the autophagy–lysosomal pathway [17]. Whether reduced degradative capacity in diseased neurons leads to LRP10 accumulation needs to be further investigated. Alternatively, LRP10-positive vesicle accumulation in LB could have a non-neuronal origin. Extensive neuron-glia coupling, including the exchange of lipids and proteins, extracellular vesicles, and mitochondria from astrocytes to neurons, have been described [34, 39, 53]. Interestingly, co-culture of PD astrocytes and unaffected control vmDANs leads to neurodegeneration and abnormal accumulation of astrocyte-derived α-synuclein [25]. Whether astrocyte-derived LRP10 can accumulate in vmDANs and cause pathology needs to be further investigated. Irrespective of the origin of the LRP10-positive vesicles in LB, our findings align with the previous observation that the LBs core consists of α-synuclein and lipid vesicle clusters [76]. Further research is required to clarify the source of LRP10 protein in neurons and the mechanisms leading to LRP10 protein accumulation in neuronal LB pathology.

In conclusion, we demonstrate that the LBD-associated protein LRP10 is expressed in the intracellular vesicle trafficking compartment of mainly astrocytes, where it can interact with the sorting receptor SORL1. Together with the finding that LRP10-positive vesicles are at the core of mature LB in substantia nigra of PD and DLB brains, this study provides further proof of an important role for LRP10 in LBD pathogenetic pathways, possibly via cell non-autonomous mechanisms. Further research is warranted to illuminate the disease mechanisms regulated by LRP10 in neurodegenerative disorders and potentially provide novel avenues for therapy development.

## Supplementary Information

Below is the link to the electronic supplementary material.

Supplementary file1 (MP4 423941 kb)

Supplementary file2 (MP4 462739 kb)

Supplementary file3 (PDF 1560 KB)
