# Supplementary material for: LRP10 interacts with SORL1 in the intracellular vesicle trafficking pathway in non-neuronal brain cells and localises to Lewy bodies in Parkinson’s disease and dementia with Lewy bodies
Source: Acta Neuropathol. 2021 Apr 28;142(1):117–37. doi: 10.1007/s00401-021-02313-3 (PMC8217053; doi:10.1007/s00401-021-02313-3)
Supplement: Supplementary file 3 — Supplementary file3 (PDF 1560 KB) [file 401_2021_2313_MOESM3_ESM.pdf]

LRP10 interacts with SORL1 in the intracellular vesicle trafficking pathway in non-neuronal brain cells and localises to Lewy bodies in Parkinson's disease and dementia with Lewy bodies

Martyna M. Grochowska[1], Ana Carreras Mascaro[1], Valerie Boumeester[1], Domenico Natale[1], Guido J. Breedveld[1], Hanneke Geut[2,3], Wiggert A. van Cappellen[4], Agnita J.W. Boon[5], Anneke J.A. Kievit[1], Esther Sammler[6,7], Netherlands Brain Bank[3], Piero Parchi[8,9], Pietro Cortelli[8,10], Dario R. Alessi[6], Wilma D.J. van de Berg[2], Vincenzo Bonifati[1], Wim Mandemakers[1]

Corresponding author's e-mail address: w.mandemakers@erasmusmc.nl

[1]Erasmus MC, University Medical Center Rotterdam, Department of Clinical Genetics, P.O. Box 2040, 3000 CA, Rotterdam, Netherlands

[2]Amsterdam Neuroscience, Amsterdam UMC, Vrije Universiteit Amsterdam, Department of Anatomy and Neurosciences, P.O. Box 7057, 1007 MB, Amsterdam, Netherlands

[3]Netherlands Institute for Neuroscience, Meibergdreef 47, 1105 BA, Amsterdam, Netherlands

[4]Erasmus MC, University Medical Center Rotterdam, Erasmus Optical Imaging Centre (OIC), P.O., Box 2040, 3000 CA, Rotterdam, the Netherlands

[5]Erasmus MC, University Medical Center Rotterdam, Department of Neurology, P.O. Box 2040, 3000 CA, Rotterdam, Netherlands

[6]Medical Research Council (MRC) Protein Phosphorylation and Ubiquitylation Unit, School of Life Sciences, University of Dundee, Dundee DD1 5EH, United Kingdom

[7]Department of Neurology, School of Medicine, University of Dundee, Ninewells Hospital, Dundee DD1 9SY, United Kingdom

[8]Istituto di Ricovero e Cura a Carattere Scientifico (IRCCS), Istituto di Scienze Neurologiche di Bologna, Via Altura 3, 40139, Bologna, Italy

[9]Department of Experimental, Diagnostic and Specialty Medicine (DIMES), University of Bologna, Via Massarenti 9, 40138, Bologna, Italy

[10]Dipartimento di Scienze Biomediche e NeuroMotorie (DIBINEM), Alma Mater Studiorum-University of Bologna, Via Altura 3, 40139, Bologna, Italy

Online Resource Material

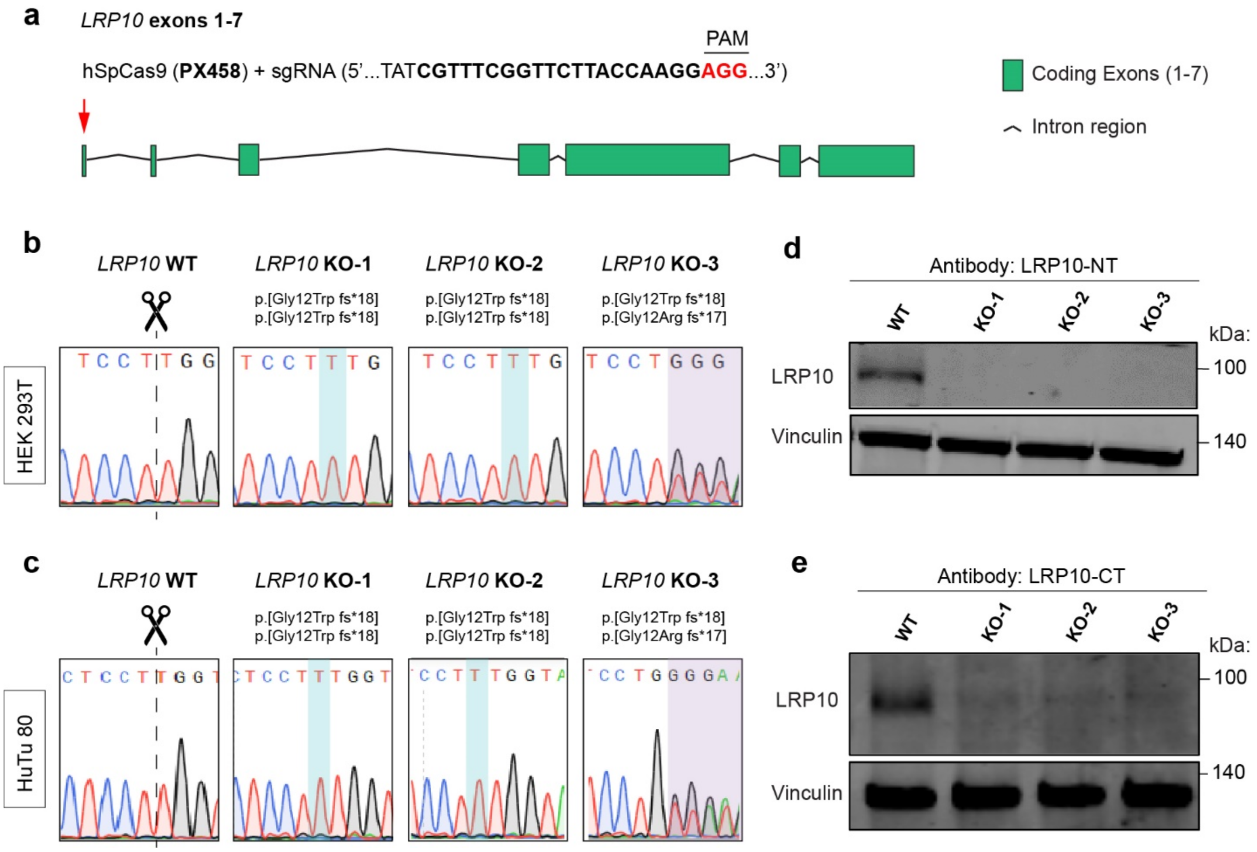

Online Resource Fig. 1 CRISPR/Cas9-mediated knockout of the *LRP10* gene. **a.** Schematic representation of a CRISPR/Cas9-mediated knockout at the *LRP10* locus. hSpCas9 together with the single guide RNA creates a double-stranded break in the first exon (red arrow). **b,c.** Electropherograms of *LRP10* wild type (WT) and different *LRP10* knockout (KO-1, KO-2, KO-3) clones generated in HEK 293T (**b**) and HuTu 80 (**c**) cell lines. Sanger sequencing confirms bi-allelic targeting in all analysed clones. All identified mutations are frameshift mutations leading to a premature stop codon (p.Gly12Trp fs*18 or p.Gly12Arg fs*17). **d.** Western blots showing endogenous LRP10 expression in LRP10 WT and KO lines using extracellular (LRP10-NT, **d**) or intracellular (LRP10-CT, **e**) LRP10 antibody for detection. Whole cell lysates were obtained from HuTu 80 clones. Vinculin was used as a loading control

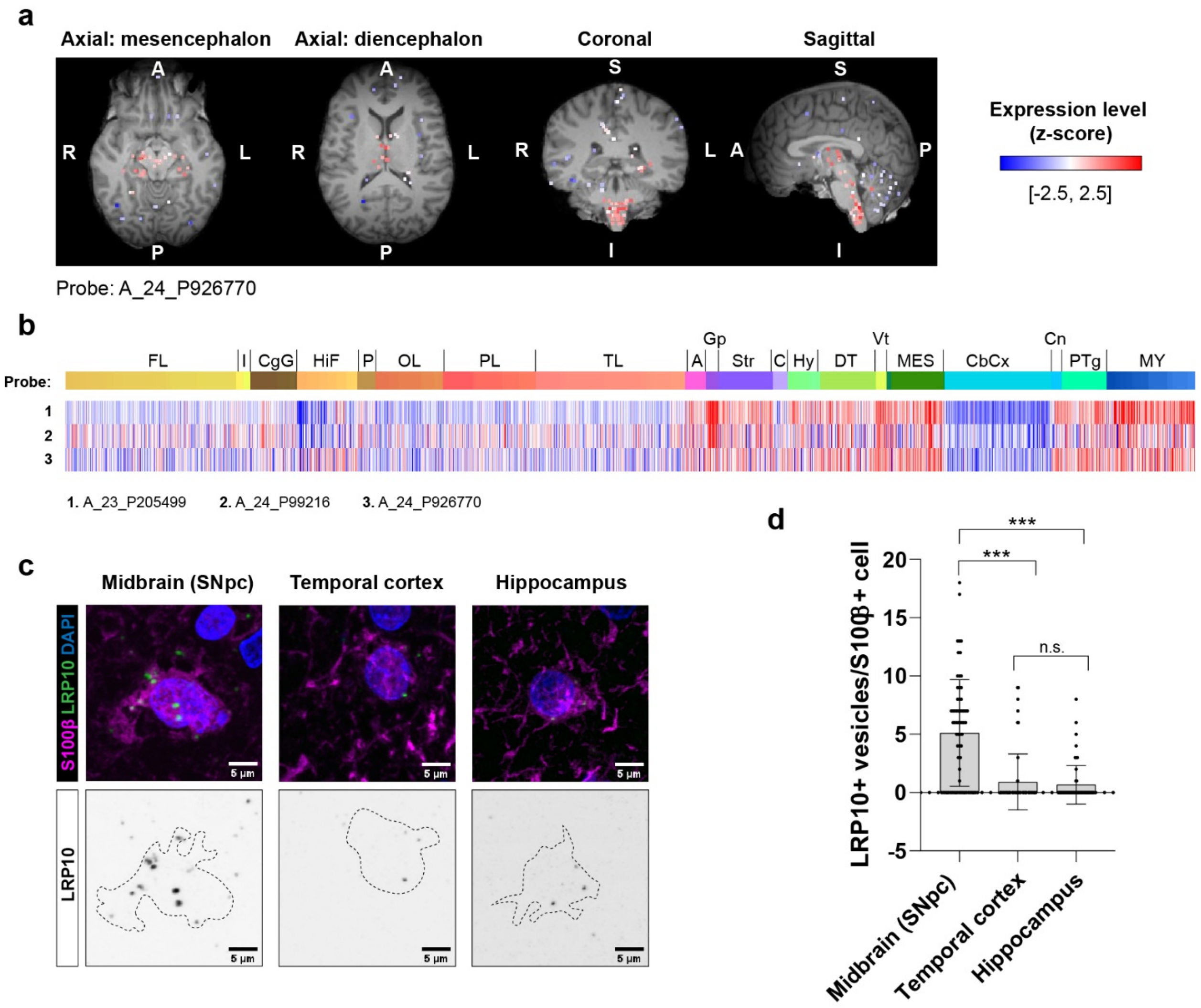

Online Resource Fig. 2 LRP10 regional expression in adult human brain. **a, b.** Microarray-based LRP10 gene expression from various brain regions. Data obtained from the Allen Human Brain Atlas. RNA microarray database (*http://human.brain-map.org/microarray/search*). Image credit: Allen Institute. **a.** Axial, coronal, and sagittal anatomical views of the LRP10 expression (z-score) in H0351.2002 donor using LRP10: A_24_P926770 probe. **b.** LRP10 expression (z-score) in various brain regions. Total RNA was isolated from 6 different brain donors; three LRP10 probes were used. R, right; L, left; A, anterior; P, posterior; I, inferior; S, superior; FL, frontal lobe; I, insula; CgG, cingulate gyrus; HiF, hippocampal formation; P, parahippocampal gyrus; OL, occipital lobe; PL, parietal lobe; TL, temporal lobe; A, amygdala; Gp, globus pallidus; Str, striatum; C, claustrum; Hy, hypothalamus; DT, dorsal thalamus; Vt,

ventral thalamus; MES, mesencephalon; CbCx, cerebellar cortex; Cn, cerebellar nuclei; PTg, pontine tegmentum; MY, myelencephalon. **c.** Astrocyte-specific LRP10 expression (green) in the midbrain (SNpc, substantia nigra pars compacta), temporal cortex, and hippocampus. Images were acquired with the same confocal microscope settings for all brain regions. **d.** Quantification of the amount of LRP10+ vesicles in S100β positive astrocytes. N=55-60 cells per brain region. Error bars represent ±SD. Kruskal-Wallis with Dunn multiple comparisons test; n.s., not significant; *** $p<0.001$. Quantifications were performed on images from a minimum of three brain sections derived from three different non-demented individuals

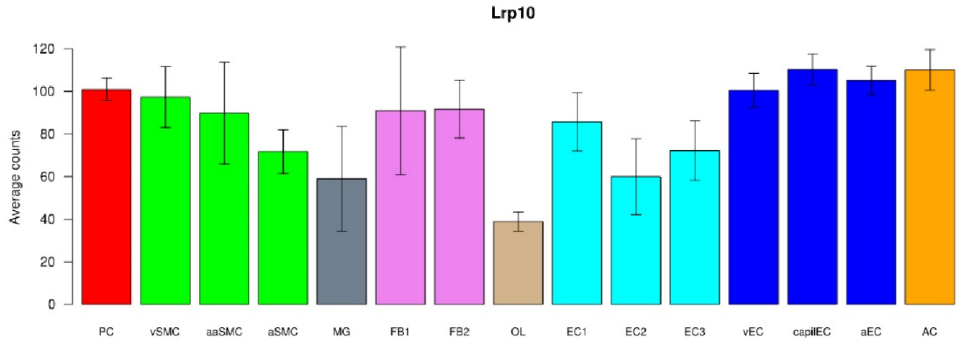

Online Resource Fig. 3 Lrp10 expression in mouse neurovasculature. Single-cell RNA sequencing of mouse brain (*http://betsholtzlab.org/VascularSingleCells/database.html*). Bars show average expression in each cell cluster. Error bars represent ±SD. PC, pericytes; SMC, smooth muscle cells; MG, microglia; FB, vascular fibroblast-like cells; OL, Oligodendrocytes; EC, endothelial cells; AC, astrocytes; v, venous; capil, capillary; a, arterial; aa, arteriolar; 1,2,3, different subtypes

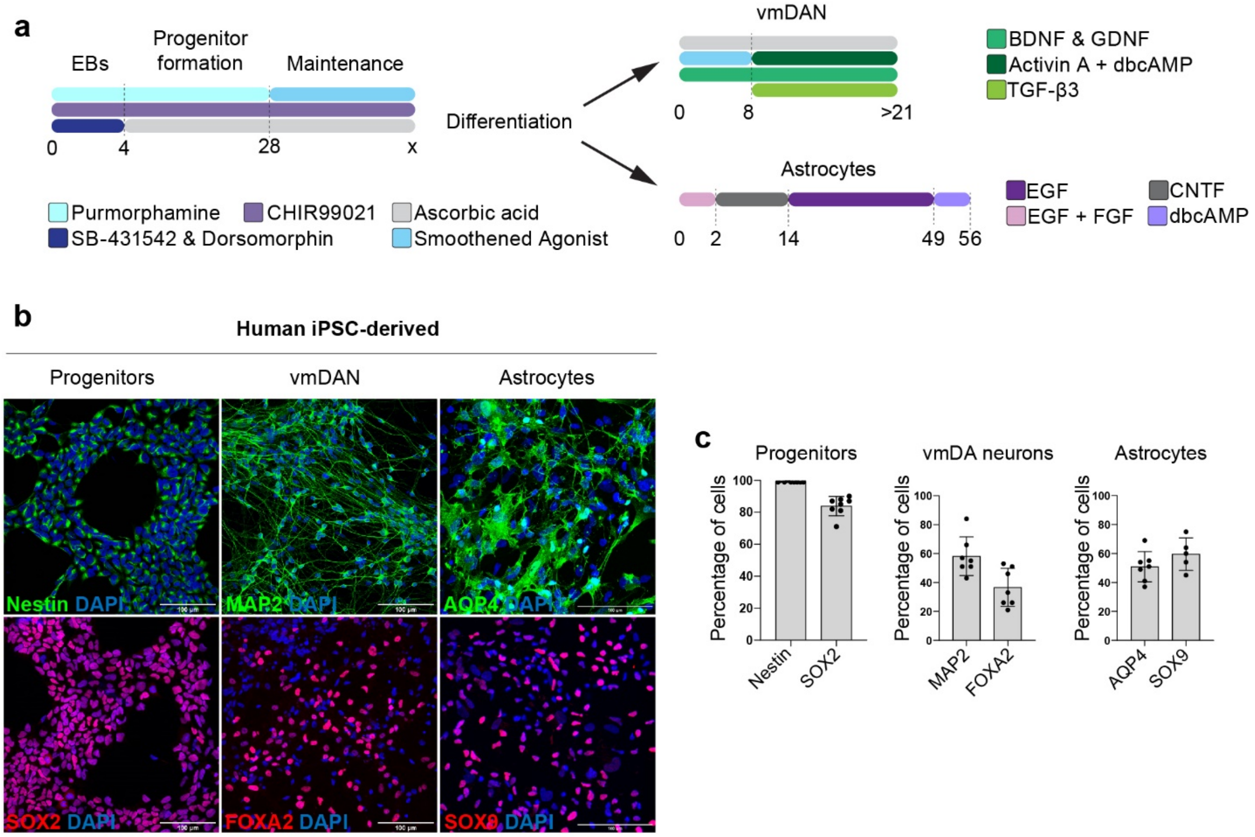

Online Resource Fig. 4 Characterisation of human iPSC-derived brain cell types. **a.** Schematic depiction of differentiation protocols used in this study. EBs, embryoid bodies; vmDAN, ventral midbrain dopaminergic neurons. **b.** Representative images of human iPSC-derived progenitors, 21 days old iPSC-derived vmDAN, and 56 days old astrocytes from a control line. Progenitors were stained with Nestin (neural progenitor marker, green) and SOX2 (pluripotency marker, red). Mature vmDAN were stained with MAP2 (dendritic marker, green) and FOXA2 (ventral midbrain-specific transcription factor, red). Astrocytes were stained with AQP4 (glial marker aquaporin 4, green) and SOX9 (astrocyte-specific nuclear marker, red). Nuclei were counterstained with DAPI (blue). Scale bars, 100 µm. **c.** Percentages of cells positive for different cell type-specific markers. Percentages were calculated to the total amount of nuclei. Data show percentages from 3 different coverslips (at least one plane of view was imaged per coverslip). Data are expressed as mean ±SD

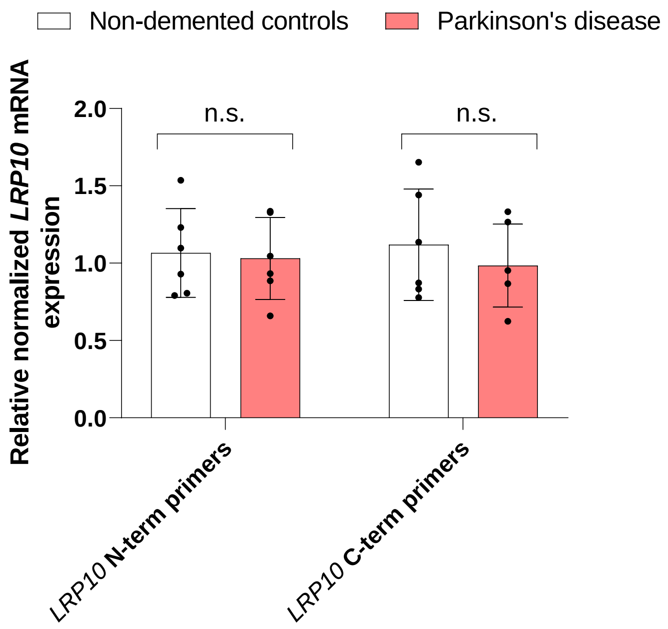

Online Resource Fig. 5 RT-qPCR analysis of the *LRP10* gene. RNA was extracted from substantia nigra of non-demented controls (N=6) and Parkinson's disease patients (N=6). Data represent relative normalized *LRP10* mRNA expression using primers designed against N-term and C-term of *LRP10* cDNA. *CLK2*, *COPS5*, *RNF10* were used as reference genes. Error bars represent ±SD. Pairwise t test; n.s., not significant. Each dot represents an individual, 6 technical replicates per individual

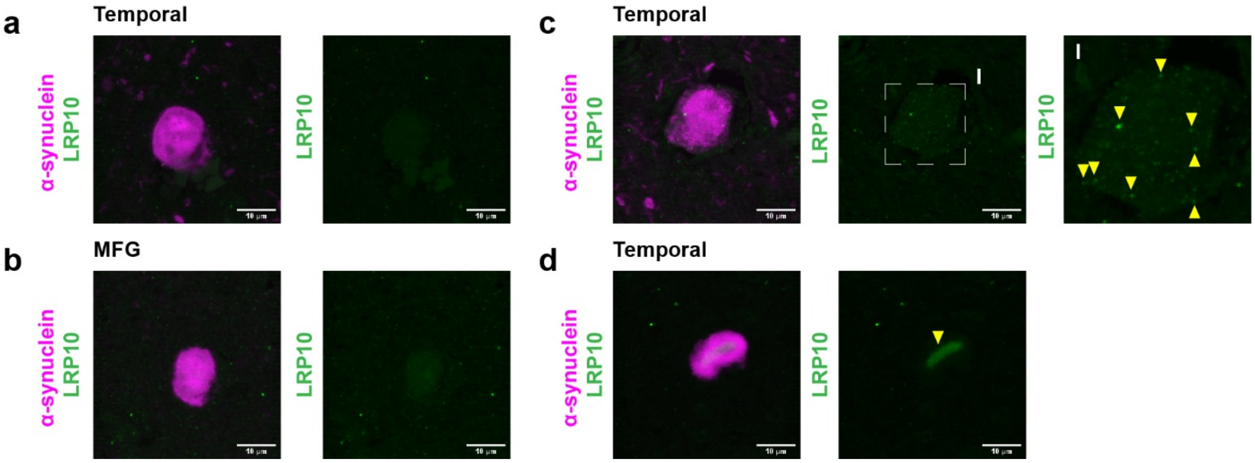

Online Resource Fig. 6 LRP10 is occasionally found in cortical LBs in the human brain. **a,b**. LRP10 (green) is absent in the majority of cortical LB (SYN-1, magenta) in the temporal cortex or medial frontal gyrus. **c.** Sporadically, LRP10 vesicles (yellow arrows) get incorporated into compact cortical LBs. **d.** LRP10 (green) immunoreactivity found in a cortical LB with targetoid appearance. Images are representative across six DLB donors and one PDD donor (*LRP10* p.Arg235Cys). Three out of six DLB cases are carriers of *LRP10* pathogenic variants (p.Arg151Cys, p.Gly453Ser, p.Asn517del). LB, Lewy body; MFG, medial frontal gyrus

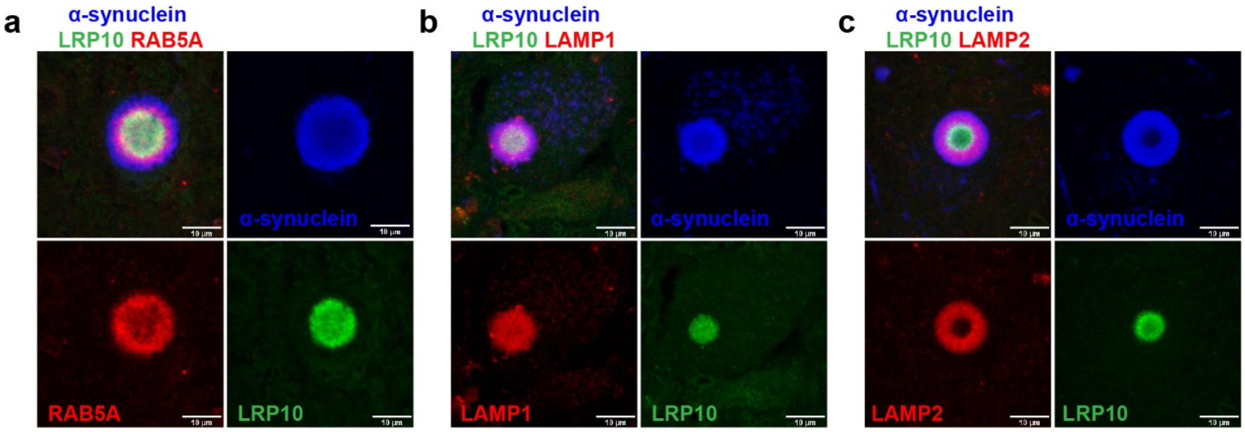

Online Resource Fig. 7 Distribution of vesicular structures within Lewy pathology. **a-c**. Distribution of LRP10 together with subcellular organellar markers detecting early endosomes, RAB5A (**a**), or lysosome-associated proteins: LAMP1 (**b**) and LAMP2 (**c**). Images are representative across 3 DLB donors. Maximum projections. Scale bars, 10 µm

Legends for the supplementary video files

Online Resource Video 1 3D volume rendering of super-resolution confocal images showing LRP10 (green) localisation in the core of a brain-stem type LB (α-synuclein, magenta) found in the substantia nigra of an idiopathic DLB patient

Online Resource Video 2 3D volume rendering of super-resolution confocal images showing LRP10 (green) localisation in the core of a brain-stem type LB (α-synuclein, magenta) found in the substantia nigra of another idiopathic DLB patient

Online Resource Table 1 Clinical and pathological characteristics of brain donors

Donor	Clinical diagnosis	Neuropathological diagnosis	Mutation status	Age at death	Age at onset	Sex	Brain weight (g)	PMD (hrs:min)	Braak NFT stage [3]	Braak α-syn stage [1]	CERAD (age-related) [4]	Thal phase [5]	CAA type	*APOE*
**NON-DEMENTED CONTROLS COHORT**														
**NDC I**	n.a.	Control	n.a.	88	n.a.	F	1186	05:45	?	1	B	n.a.	n.a.	3/3
**NDC II**	n.a.	Control	n.a.	62	n.a.	M	1350	06:35	0	n.a.	n.a.	n.a.	n.a.	4/3
**NDC III**	n.a.	Control	n.a.	87	n.a.	F	1095	07:20	3	n.a.	O	n.a.	n.a.	n.a.
**NDC IV**	n.a.	Control	n.a.	78	n.a.	M	1125	17:40	1	n.a.	O	n.a.	n.a.	3/3
**NDC V**	n.a.	Control	n.a.	60	n.a.	F	1215	05:30	0	0	O	1	2	n.a.
**NDC VI**	n.a.	Control	n.a.	98	n.a.	M	1153	08:40	1	n.a.	B	n.a.	n.a.	3/3
**NDC VII**	n.a.	Control	n.a.	82	n.a.	F	1175	11:30	1	0	O	0	0	3/3
**NDC VIII**	n.a.	Control	n.a.	89	n.a.	F	1155	06:25	2	0	A	n.a.	0	3/2
**NDC IX**	n.a.	Control	n.a.	84	n.a.	F	1216	06:55	1	0	O	0	n.a.	n.a.
**NDC X**	n.a.	Control	n.a.	95	n.a.	F	1010	07:05	3	0	A	3	n.a.	n.a.
**NDC XI**	n.a.	Control	n.a.	82	n.a.	M	1195	05:45	2	0	O	3	0	n.a.
**LRP10 VARIANT CARRIERS COHORT**														
**Patient I**	AD*	DLB	c.451C>T; p.Arg151Cys	90	80	F	1053	05:35	4	6	B	3	2	3/3
**Patient II**	PD†	LBD	c.632dupT; p.Ala212Ser fs*17	90	74	F	1335	04:45	3	6	A	3	2	3/3
**Patient III**	PDD†	LBD	c.703C>T; p.Arg235Cys;	82	74	F	n.a.	n.a.	3	6	A	n.a.	n.a.	3/3
**Patient IV**	DLB**	Mixed AD/LBD	c.1357G>A; p.Gly453Ser	72	64	F	1043	05:35	6	6	C	5	2	3/4
**Patient V**	DLB†	LBD	c.1549_1551delAAT; p.Asn517del	79	74	M	1270	04:25	4	6	A	3	1	4/3
**PARKINSON'S DISEASE COHORT**														
**PD I**	PDD	PDD	n.a.	87	n.a.	M	1343	04:45	2	5	A	3	2	3/3
**PD II**	PD	PD	n.a.	88	n.a.	F	1130	04:15	0	6	O	1	2	n.a.
**PD III**	PD	PD	n.a.	59	n.a.	F	1465	09:35	1	5	O	1	0	n.a.
**PD IV**	PD	PD	n.a.	81	n.a.	F	1145	06:40	1	6	C	3	2	n.a.
**PD V**	PD	PD	n.a.	90	n.a.	F	1155	05:20	3	6	O	1	0	n.a.
**PD VI**	PDD	PDD	n.a.	68	n.a.	F	1190	07:15	3	6	O	0	0	n.a.
**PD VII**	PD	PD	n.a.	86	n.a.	M	1215	04:50	2	4	A	3	1	n.a.
**PD VIII**	PD	PD	n.a.	80	n.a.	M	1505	06:00	2	6	O	0	0	n.a.
**PD IX**	PD	PD	n.a.	92	n.a.	M	1245	08:50	3	3	O	3	n.a.	n.a.
**DEMENTIA WITH LEWY BODIES COHORT**														
**DLB I**	DLB	DLB + AD	n.a.	81	n.a.	F	1118	07:05	3	5	A	4	2	4/3
**DLB II**	DLB	DLB	n.a.	79	n.a.	M	1316	04:00	1	6	A	2	1	n.a.
**DLB III**	DLB	DLB	n.a.	80	n.a.	F	1231	05:00	1	6	O	0	0	n.a.

LBD, Lewy body disease; *, clinical diagnosis AD, neuropathological diagnosis DLB; **, clinical diagnosis DLB, neuropathological diagnosis mixed AD/LBD; †, neuropathological diagnosis LBD; Mean age at death±SD: 81.39, ±9.54; 64% females; PMD, post mortem delay; NFT,

neurofibrillary tangles; α-syn, α-synuclein; CERAD, Consortium to Establish a Registry for Alzheimer Disease; *APOE*, apolipoprotein E; NDC, non-demented controls; AD, Alzheimer's disease; PD, Parkinson's disease; PDD, Parkinson's disease dementia; DLB, dementia with Lewy bodies; M, male; F, female; Age, age at death; n.a., information not available; ?, uncertain

Online Resource Table 2 Details of the primary antibodies used in this study

#Catalogue	Source	Epitope	Host	Application
**13228-T16**	Sino Biological	LRP10-NT (1-440 aa)	Rabbit	WB 1:500<br>ICC 1:100<br>IHC-P 1:100<br>IHC-Fr 1:100
**DA058**	MRC PPU Reagents	LRP10-CT (463-713 aa)	Sheep	WB 1:500<br>ICC 1:100<br>IHC-P 1:100
**AB5603**	Millipore Sigma	SOX2	Rabbit	ICC 1:200
**MAB5326**	Millipore Sigma	Nestin (10C2)	Mouse	ICC 1:200
**188 004**	Synaptic Systems	MAP2	Guinea pig	ICC 1:250<br>IHC-P 1:250
**8186**	Cell Signalling Technology	FoxA2/HNF3β (D56D6)	Rabbit	ICC 1:200
**HPA014784**	Atlas Antibodies	AQP4	Rabbit	ICC 1:200<br>IHC-P 1:500
**AF3075**	R&D Systems	SOX9	Goat	ICC 1:100
**NB300-110**	Novus Biologicals	TH	Sheep	ICC 1:100<br>IHC-P 1:100
**S2532**	Sigma-Aldrich	S100β	Rabbit	ICC 1:100<br>IHC-P 1:100
**HPA015768**	Sigma-Aldrich	S100β	Mouse	ICC 1:100<br>IHC-P 1:100
**019-19741**	FUJIFILM Wako Chemicals	Iba1	Rabbit	IHC-P 1:500
**ab16794**	Abcam	APC (CC1), Quaking 7 [2]	Mouse	IHC-P 1:200
**ab7817**	Abcam	α-smooth muscle actin (1A4)	Mouse	IHC-P 1:500
**M076101-2**	Agilent Dako	GFAP (6F2)	Mouse	IHC-P 1:200
**AHP500GT**	Bio-Rad	TGN46	Sheep	ICC 1:500
**610457**	BD Biosciences	EEA1	Mouse	ICC 1:200
**ab10099**	Abcam	VPS35	Goat	ICC 1:100
**SAB4700416**	Millipore Sigma	LAMP1 (H4A3)	Mouse	ICC 1:100<br>IHC 1:250
**MABN1793**	Merck	SorLA (20C11)	Mouse	ICC 1:100<br>IHC-Fr 1:100
**612633**	BD Biosciences	LR11/SorLA	Mouse	WB 1:1000<br>ICC 1:100<br>IHC-Fr 1:100
**610786**	BD Biosciences	α-synuclein (SYN-1)	Mouse	IHC-P 1:100
**ab209538**	Abcam	α-synuclein (MJF-14-6-4-2)	Rabbit	IHC 1:500
**11947-1-AP**	Proteintech	RAB5A	Rabbit	IHC-P 1:100
**ab18528**	Abcam	LAMP2A	Rabbit	IHC-P 1:100
**sc-59803**	Santa Cruz Biotechnology	Vinculin (V284)	Mouse	WB 1:3000

WB, Western blot; ICC, immunocytochemistry; IHC, immunohistochemistry; P, formalin-fixed, paraffin-embedded; Fr, fresh frozen

Online Resource Table 3 RT-qPCR primer sequences

Name	Sequence
	LRP10 primers
LRP10-N-terminal-FW	ACTGCACCTGGCTCATCC
LRP10-N-terminal-RV	GAGATCAGTGGCTGGAGAGG
LRP10-C-terminal-FW	CCAGGAGTACAGCATCTTTGC
LRP10-C-terminal-RV	TAGCAGAGAACGCAGGTTGC
	SNCA primers
SNCA-N-terminal-FW	AGCCATGGATGTATTCATGAAAGG
SNCA-N-terminal-RV	TTGGAGCCTACATAGAGAACACCC
SNCA-C-terminal-FW	TGGGCAAGAATGAAGAAGGAGC
SNCA-C-terminal-RV	GGTTCGTAGTCTTGATACCCTTCC
	Reference genes
RNF10-FW	GCATCTGTGAACTGGCTTTG
RNF10-RV	CTGACGTTTCCTCTTCTCAATG
CLK2-FW	TCGTTAGCACCTTAGGAGAGG
CLK2-RV	TGATCTTCAGGGCAACTCG
COPS5-FW	CCAGGAACCATTTGTAGCAG
COPS5-RV	GTAGCCCTTTGGGTATGTCC

Online Resource Table 4 Commercial LRP10 antibodies screened in this study

#Catalogue	Company	Species	Immunogen range (amino acids)	Clonality
ABP54460	Abbkine	Rabbit	180-260	Polyclonal
PAB17761	Abnova	Rabbit	Internal part, not specified	Polyclonal
sc-243324	Santa Cruz Biotechnology	Goat	Extracellular domain, not specified	Polyclonal
HPA000932	Sigma-Aldrich	Rabbit	24-161	Polyclonal
SAB4501107	Sigma-Aldrich	Rabbit	204-253	Polyclonal
13228-T16	Sino Biological	Rabbit	1-440	Polyclonal
PA5-39229	Invitrogen	Rabbit	Internal part, not specified	Polyclonal

References

1 Alafuzoff I, Ince PG, Arzberger T, Al-Sarraj S, Bell J, Bodi I, Bogdanovic N, Bugiani O, Ferrer I, Gelpi Eet al (2009) Staging/typing of Lewy body related α-synuclein pathology: a study of the BrainNet Europe Consortium. Acta Neuropathologica 117: 635-652 Doi 10.1007/s00401-009-0523-2

2 Bin JM, Harris SN, Kennedy TE (2016) The oligodendrocyte-specific antibody 'CC1' binds Quaking 7. J Neurochem 139: 181-186

3 Braak H, Alafuzoff I, Arzberger T, Kretzschmar H, Del Tredici K (2006) Staging of Alzheimer disease-associated neurofibrillary pathology using paraffin sections and immunocytochemistry. Acta Neuropathologica 112: 389-404 Doi 10.1007/s00401-006-0127-z

4 Hyman BT, Phelps CH, Beach TG, Bigio EH, Cairns NJ, Carrillo MC, Dickson DW, Duyckaerts C, Frosch MP, Masliah Eet al (2012) National Institute on Aging-Alzheimer's Association guidelines for the neuropathologic assessment of Alzheimer's disease. Alzheimer's & dementia : the journal of the Alzheimer's Association 8: 1-13 Doi 10.1016/j.jalz.2011.10.007

5 Thal DR, Rüb U, Orantes M, Braak H (2002) Phases of A beta-deposition in the human brain and its relevance for the development of AD. Neurology 58: 1791-1800
